# Genomics of perivascular space burden unravels early mechanisms of cerebral small vessel disease

**DOI:** 10.1038/s41591-023-02268-w

**Published:** 2023-04-17

**Authors:** Marie-Gabrielle Duperron, Maria J. Knol, Quentin Le Grand, Tavia E. Evans, Aniket Mishra, Ami Tsuchida, Gennady Roshchupkin, Takahiro Konuma, David-Alexandre Trégouët, Jose Rafael Romero, Stefan Frenzel, Michelle Luciano, Edith Hofer, Mathieu Bourgey, Nicole D. Dueker, Pilar Delgado, Saima Hilal, Rick M. Tankard, Florian Dubost, Jean Shin, Yasaman Saba, Nicola J. Armstrong, Constance Bordes, Mark E. Bastin, Alexa Beiser, Henry Brodaty, Robin Bülow, Caty Carrera, Christopher Chen, Ching-Yu Cheng, Ian J. Deary, Piyush G. Gampawar, Jayandra J. Himali, Jiyang Jiang, Takahisa Kawaguchi, Shuo Li, Melissa Macalli, Pascale Marquis, Zoe Morris, Susana Muñoz Maniega, Susumu Miyamoto, Masakazu Okawa, Matthew Paradise, Pedram Parva, Tatjana Rundek, Muralidharan Sargurupremraj, Sabrina Schilling, Kazuya Setoh, Omar Soukarieh, Yasuharu Tabara, Alexander Teumer, Anbupalam Thalamuthu, Julian N. Trollor, Maria C. Valdés Hernández, Meike W. Vernooij, Uwe Völker, Katharina Wittfeld, Tien Yin Wong, Margaret J. Wright, Junyi Zhang, Wanting Zhao, Yi-Cheng Zhu, Helena Schmidt, Perminder S. Sachdev, Wei Wen, Kazumichi Yoshida, Anne Joutel, Claudia L. Satizabal, Ralph L. Sacco, Guillaume Bourque, the CHARGE consortium, Quentin Le Grand, Mark Lathrop, Tomas Paus, Israel Fernandez-Cadenas, Qiong Yang, Bernard Mazoyer, Philippe Boutinaud, Yukinori Okada, Hans J. Grabe, Karen A. Mather, Reinhold Schmidt, Marc Joliot, M. Arfan Ikram, Fumihiko Matsuda, Christophe Tzourio, Joanna M. Wardlaw, Sudha Seshadri, Hieab H. H. Adams, Stéphanie Debette

**Affiliations:** 1 Bordeaux Population Health Research Center, UMR 1219, University of Bordeaux, Inserm, Bordeaux, France; 2 Department of Neurology, Institute of Neurodegenerative Diseases, Bordeaux University Hospital, Bordeaux, France; 3 Department of Epidemiology, Erasmus MC University Medical Center, Rotterdam, the Netherlands; 4 Department of Clinical Genetics, Erasmus MC University Medical Center, Rotterdam, the Netherlands; 5 Department of Radiology and Nuclear Medicine, Erasmus MC University Medical Center, Rotterdam, the Netherlands; 6 Groupe d’Imagerie Neurofonctionelle - Institut des maladies neurodégénératives (GIN-IMN), UMR 5293, University of Bordeaux, CNRS, CEA, Bordeaux, France; 7 Department of Statistical Genetics, Osaka University Graduate School of Medicine, Suita, Japan; 8 Department of Neurology, Boston University School of Medicine, Boston, MA USA; 9 The Framingham Heart Study, Framingham, MA USA; 10 Department of Psychiatry and Psychotherapy, University Medicine Greifswald, Greifswald, Germany; 11 School of Psychology, University of Edinburgh, Edinburgh, UK; 12 Clinical Division of Neurogeriatrics, Department of Neurology, Medical University of Graz, Graz, Austria; 13 Institute for Medical Informatics, Statistics and Documentation, Medical University of Graz, Graz, Austria; 14 Department of Human Genetics, McGill University, Montreal, Quebec Canada; 15 Victor Phillip Dahdaleh Institute of Genomic Medicine at McGill University, Montreal, Quebec Canada; 16 Canadian Centre for Computational Genomics, McGill University, Montreal, Quebec Canada; 17 John P. Hussman Institute for Human Genomics, University of Miami, Miami, FL USA; 18 Institut de Recerca Vall d’hebron, Neurovascular Research Lab, Universitat Autònoma de Barcelona, Barcelona, Spain; 19 Hospital Universitari Vall d’Hebron, Neurology Department, Universitat Autònoma de Barcelona, Barcelona, Spain; 20 Memory Aging and Cognition Center, National University Health System, Singapore, Singapore; 21 Department of Pharmacology, Yong Loo Lin School of Medicine, National University of Singapore, Singapore, Singapore; 22 Saw Swee Hock School of Public Health, National University of Singapore and National University Health System, Singapore, Singapore; 23 Department of Mathematics and Statistics, Curtin University, Perth, Western Australia Australia; 24 Department of Medical Informatics, Erasmus MC University Medical Center, Rotterdam, the Netherlands; 25 The Hospital for Sick Children, University of Toronto, Toronto, Ontario Canada; 26 Departments of Physiology and Nutritional Sciences, University of Toronto, Toronto, Ontario Canada; 27 Institute for Molecular Biology & Biochemistry, Gottfried Schatz Research Center (for Cell Signaling, Metabolism and Aging), Medical University of Graz, Graz, Austria; 28 Centre for Clinical Brain Sciences, University of Edinburgh, Edinburgh, UK; 29 Department of Biostatistics, Boston University School of Public Health, Boston, MA USA; 30 Centre for Healthy Brain Ageing (CHeBA), Discipline of Psychiatry & Mental Health, University of New South Wales, Sydney, New South Wales Australia; 31 Dementia Collaborative Research Centre Assessment and Better Care, UNSW, Sydney, New South Wales Australia; 32 Institute for Radiology and Neuroradiology, University Medicine Greifswald, Greifswald, Germany; 33 Stroke Pharmacogenomics and Genetics Group, Biomedical Research Institute Sant Pau (IIB Sant Pau), Barcelona, Spain; 34 Department of Psychological Medicine, Yong Loo Lin School of Medicine, National University of Singapore, Singapore, Singapore; 35 Singapore Eye Research Institute, Singapore National Eye Centre, Singapore, Singapore; 36 Center for Innovation and Precision Eye Health, Yong Loo Lin School of Medicine, National University of Singapore, Singapore, Singapore; 37 Department of Ophthalmology, Yong Loo Lin School of Medicine, National University of Singapore, Singapore, Singapore; 38 Ophthalmology & Visual Sciences Academic Clinical Program, Duke-NUS Medical School, Singapore, Singapore; 39 Glenn Biggs Institute for Alzheimer’s and Neurodegenerative Diseases, UT Health San Antonio, San Antonio, TX USA; 40 Department of Population Health Sciences, UT Health San Antonio, San Antonio, TX USA; 41 Center for Genomic Medicine, Kyoto University Graduate School of Medicine, Kyoto, Japan; 42 Neuroimaging, Department of Clinical Neurosciences, Royal Infirmary of Edinburgh, Edinburgh, UK; 43 UK Dementia Research Institute Centre at the University of Edinburgh, Edinburgh, UK; 44 Kyoto University Hospital, Kyoto, Japan; 45 Department of Neurosurgery, Kyoto University Graduate School of Medicine, Kyoto, Japan; 46 Radiology Department, Boston University School of Medicine, Boston, MA USA; 47 Department of Radiology, Harvard Medical School, Boston, MA USA; 48 Department of Neurology, Miller School of Medicine, University of Miami, Miami, FL USA; 49 Evelyn F. McKnight Brain Institute, Department of Neurology, University of Miami, Miami, FL USA; 50 Graduate School of Public Health, Shizuoka Graduate University of Public Health, Shizuoka, Japan; 51 Institute for Community Medicine, University Medicine Greifswald, Greifswald, Germany; 52 Department of Developmental Disability Neuropsychiatry, UNSW, Sydney, New South Wales Australia; 53 Row Fogo Centre for Research into Ageing and the Brain, University of Edinburgh, Edinburgh, UK; 54 Interfaculty Institute for Genetics and Functional Genomics, University Medicine Greifswald, Greifswald, Germany; 55 German Center for Neurodegenerative Diseases (DZNE), Site Rostock/Greifswald, Greifswald, Germany; 56 Tsinghua Medicine, Tsinghua University, Beijing, China; 57 Queensland Brain Institute, University of Queensland, Brisbane, Queensland Australia; 58 Centre for Advanced Imaging, University of Queensland, Brisbane, Queensland Australia; 59 Department of Neurology, Peking Union Medical College Hospital, Beijing, China; 60 The Centre for Quantitative Medicine, Duke-NUS Medical School, Singapore, Singapore; 61 Neuropsychiatric Institute, the Prince of Wales Hospital, Sydney, New South Wales Australia; 62 Institut de Psychiatrie et Neurosciences de Paris, Université Paris Cité, Inserm, France; 63 Department of Public Health Sciences, Miller School of Medicine, University of Miami, Miami, FL USA; 64 Department of Human Genomics, Miller School of Medicine, University of Miami, Miami, FL USA; 65 Department of Neurosurgery, Miller School of Medicine, University of Miami, Miami, FL USA; 66 University of Montreal, Faculty of Medicine, Departments of Psychiatry and Neuroscience, Montreal, Quebec Canada; 67 Department of Psychology, University of Toronto, Toronto, Ontario Canada; 68 Centre Hospitalier Universitaire Sainte Justine, Montreal, Quebec Canada; 69 Department of Psychiatry, University of Toronto, Toronto, Ontario Canada; 70 Stroke Pharmacogenomics and Genetics Group, Fundació per la Docència i la Recerca Mutua Terrassa, Terrassa, Spain; 71 Bordeaux University Hospital, Bordeaux, France; 72 Fealinx, Lyon, France; 73 Laboratory of Statistical Immunology, Immunology Frontier Research Center (WPI-IFReC), Osaka University, Suita, Japan; 74 Integrated Frontier Research for Medical Science Division, Institute for Open and Transdisciplinary Research Initiatives, Osaka University, Suita, Japan; 75 Center for Infectious Disease Education and Research (CiDER), Osaka University, Suita, Japan; 76 Department of Genome Informatics, Graduate School of Medicine, University of Tokyo, Tokyo, Japan; 77 Neuroscience Research Australia, Sydney, New South Wales Australia; 78 Department of Medical Informatics, Bordeaux University Hospital, Bordeaux, France; 79 Latin American Brain Health (BrainLat), Universidad Adolfo Ibáñez, Santiago, Chile

**Keywords:** Neurology, Stroke, Genome-wide association studies

## Abstract

Perivascular space (PVS) burden is an emerging, poorly understood, magnetic resonance imaging marker of cerebral small vessel disease, a leading cause of stroke and dementia. Genome-wide association studies in up to 40,095 participants (18 population-based cohorts, 66.3 ± 8.6 yr, 96.9% European ancestry) revealed 24 genome-wide significant PVS risk loci, mainly in the white matter. These were associated with white matter PVS already in young adults (*N* = 1,748; 22.1 ± 2.3 yr) and were enriched in early-onset leukodystrophy genes and genes expressed in fetal brain endothelial cells, suggesting early-life mechanisms. In total, 53% of white matter PVS risk loci showed nominally significant associations (27% after multiple-testing correction) in a Japanese population-based cohort (*N* = 2,862; 68.3 ± 5.3 yr). Mendelian randomization supported causal associations of high blood pressure with basal ganglia and hippocampal PVS, and of basal ganglia PVS and hippocampal PVS with stroke, accounting for blood pressure. Our findings provide insight into the biology of PVS and cerebral small vessel disease, pointing to pathways involving extracellular matrix, membrane transport and developmental processes, and the potential for genetically informed prioritization of drug targets.

## Abstract (web summary)

Genomic analyses of large population-based cohorts uncover the genetic determinants of perivascular space burden, an MRI marker of cerebral small vessel disease, across the lifespan, and reveal potential pathways implicated in the etiology of stroke and dementia.

## Main

PVS are physiological spaces surrounding small vessel walls as they run from the subarachnoid space through the brain parenchyma^1–3^. Dilation of PVS observed on brain magnetic resonance imaging (MRI) is thought to be a marker of PVS dysfunction and, speculated from preclinical studies, to reflect impairment of brain fluid and waste clearance^2,4^.

PVS increase in number with age and vascular risk factors, especially hypertension^2^. They are associated with white matter hyperintensities (WMH) of presumed vascular origin, lacunes and cerebral microbleeds^2^, all MRI features of cerebral small vessel disease (cSVD), a leading cause of stroke and dementia with no specific mechanistic treatment to date^5,6^. PVS are detected on brain MRI much earlier than WMH, lacunes or cerebral microbleeds^7^, and are described as the earliest stage of cSVD lesions on neuropathology^8^. Their pathophysiology is poorly understood^6,9^.

In experimental models, PVS appear to be important conduits for substrate delivery, flushing interstitial fluid, clearing metabolic waste (for example, beta-amyloid peptide) and brain fluid regulation, as part of the ‘glymphatic system’^4,7^. These processes were described to increase during sleep^2,4,7^. Mounting evidence suggests a major role of PVS in cerebral injury. Several studies suggested associations of PVS burden (number of visible PVS on brain MRI) with stroke^2,6,10^, Alzheimer’s disease pathology^2^ and cerebral amyloid angiopathy (CAA)^11–13^. Post-stroke edema has been linked to post-stroke PVS enlargement^14^, and in amyotrophic lateral sclerosis PVS dilation was observed and perivascular fibroblast proteins were associated with survival^15^.

PVS burden is highly heritable^16^. Identifying genetic risk variants for PVS could be a powerful tool to decipher underlying biological pathways. We conducted genome-wide association study (GWAS) meta-analyses and whole-exome/whole-genome sequencing (WES/WGS) studies of extensive PVS burden in up to 40,095 and 19,010 older community participants, respectively. Given differential associations with risk factors and neurological traits^2,10,17^ and anatomical differences^18^, we ran analyses separately for white matter (WM)-PVS, basal ganglia (BG)-PVS and hippocampal (HIP)-PVS. We followed up identified risk loci in independent samples of young healthy adults and older Japanese community participants and examined shared genetic determinants with other vascular and neurological traits. Leveraging tissue and cell-specific gene expression databases and drug target libraries, we conducted extensive bioinformatics exploration of identified PVS risk loci.

## Results

### Genetic discovery

Twenty-one population-based cohorts were included, of which 18 were for GWAS and 8 for whole-exome association studies (Supplementary Table 1 and Methods). We tested associations of extensive PVS burden with ~8 million single-nucleotide polymorphisms (SNPs) (minor allele frequency (MAF) ≥ 1%) in GWAS meta-analyses, gathering up to 40,095 participants (66.3 ± 8.6 yr, 51.7% female, 66.7% with hypertension; Supplementary Tables 1–3). We dichotomized PVS burden based on cut-offs closest to the top quartile of PVS distribution to account for differences in PVS quantification methods, image acquisition and participant characteristics (Supplementary Tables 1 and 2 and Methods). In total, 9,607 of 39,822, 9,189 of 40,000, and 9,339 of 40,095 participants had extensive PVS burden in WM, BG and hippocampus.

**Fig. 1:**
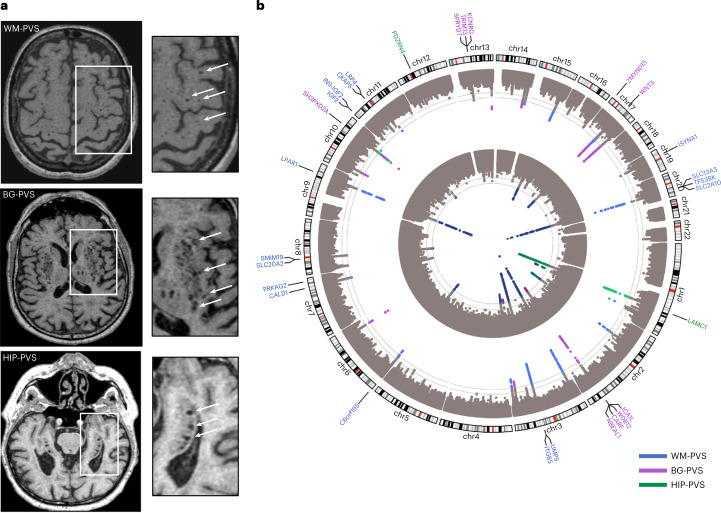
Illustration of extensive PVS burden and results of the cross-ancestry PVS GWAS meta-analysis, MTAG analysis and gene-based test. **a**, Extensive PVS burden (arrows) in WM (top, WM-PVS), BG (middle, BG-PVS) and hippocampus (bottom, HIP-PVS) on T1-weighted axial magnetic resonance images. **b**, Circular Manhattan plot. The inner circle corresponds to the cross-ancestry GWAS meta-analyses results, the middle circle to the results of the MTAG analysis and the outer circle to gene-based test results. Results for WM-PVS are in blue, for BG-PVS in purple and for HIP-PVS in green. The gray line corresponds to the genome-wide significance threshold (*P* = 5 × 10^−8^, two-sided, correcting for multiple testing at the genome-wide level).

**Extended Data Fig. 1:**
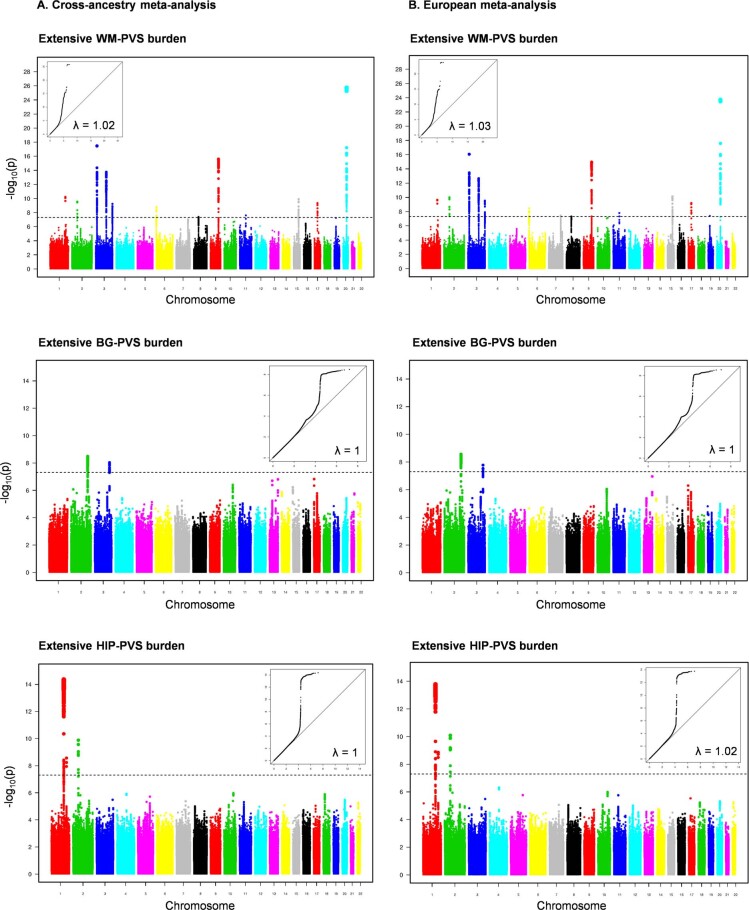
Manhattan and QQ plots of extensive PVS burden in the cross-ancestry and European meta-analyses. Manhattan and Quantile-Quantile (QQ) plots of the p-values (observed versus expected) in the cross-ancestry (A) and European (B) GWAS meta-analyses are presented along with the genomic inflation factor (λ). For QQ plots the observed -log_10_(p) is represented in the y-axis and the expected -log_10_(p) in x-axis. The dotted line corresponds to the genome-wide significance threshold (p = 5×10^−8^, two sided). PVS indicates perivascular spaces; WM, white matter; BG, basal ganglia; HIP, hippocampus.

The GWAS meta-analysis comprised 17 cohorts from the Cohorts for Heart and Aging Research in Genomic Epidemiology (CHARGE) consortium (*N* ≤ 11,511)^19^, with PVS quantification primarily on visual rating scales, and UK Biobank (UKB, *N* ≤ 28,655), with computational PVS quantification (Methods). Participants were of European (*N* = 38,871), Hispanic (*N* = 717), East-Asian (*N* = 339) and African-American (*N* = 168) ancestry. We identified 22 independent genome-wide significant risk loci for extensive PVS burden (WM-PVS: 19; BG-PVS: 2; HIP-PVS: 3 (2 shared with WM-PVS)) and two additional risk loci for WM-PVS in Europeans only, leading to 24 independent signals (Table 1, Fig. 1, Extended Data Fig. 1 and Supplementary Fig. 1). There was no systematic inflation of association statistics (Supplementary Table 4 and Extended Data Fig. 1).

**Table 1:** Genetic variants associated with high PVS burden

Region	SNP ALL	chr:position	EA/OA	EAF	Function	Nearest gene(s)	Effect (β)^a^	SE^a^	*Z*-score^b^	Dir^b^	*N* ext-PVS/*N* total	*P* value EUR	*P* value All	Het *P* value
**PVS in white matter (WM-PVS)**														
20q13.12	rs6011998	20:45269867	C/T	0.95	intronic	*SLC13A3*	0.087	0.009	10.65	++++	9,502/39,128	**1.90** **×** **10**^**−24**^	**1.80** **×** **10**^**−26**^	0.11
3p25.1	rs13079464	3:13822439	C/G	0.46	intergenic	*WNT7A*	0.026	0.004	8.70	++++	9,614/39,822	**8.64** **×** **10**^**−17**^	**3.41** **×** **10**^**−18**^	0.59
20q13.12	rs2425884	20:45258292	C/T	0.57	intronic	*SLC13A3*	0.029	0.004	8.63	+−+−	9,614/39,822	**2.60** **×** **10**^**−18**^	**6.02** **×** **10**^**−18**^	0.14
9q31.3	rs10817108^c^	9:113658671	A/G	0.21	intronic	*LPAR1*	0.029	0.004	8.20	+++?	9,550/39,516	**1.07** **×** **10**^**−15**^	**2.46** **×** **10**^**−16**^	0.75
20q13.12	rs2425881	20:45255618	A/G	0.83	intronic	*SLC13A3*	0.033	0.005	7.68	+−+?	9,496/39,087	**2.02** **×** **10**^**−15**^	**1.59** **×** **10**^**−14**^	0.06
3q21.2	rs3772833	3:124518362	G/A	0.83	intronic	*ITGB5*, *UMPS*	0.032	0.005	7.67	+++?	9,496/39,087	**2.15** **×** **10**^**−13**^	**1.76** **×** **10**^**−14**^	0.39
20q13.12	rs112407396	20:45276381	T/A	0.03	intronic	*SLC13A3*	0.078	0.012	6.91	+???	8,426/34,530	**4.81** **×** **10**^**−12**^	**4.81** **×** **10**^**−12**^	1.00
1q41	rs10494988	1:215141570	C/T	0.63	intergenic	*CENPF*, *KCNK2*	0.021	0.004	6.54	++++	9,614/39,822	**2.23** **×** **10**^**−10**^	**6.03** **×** **10**^**−11**^	0.69
20q13.12	rs72485816^d^	20:45314435	T/C	0.96	UTR3	*TP53RK*, *SLC13A3*	0.059	0.010	6.45	++?−	9,114/37,342	**1.47** **×** **10**^**−10**^	**1.12** **×** **10**^**−10**^	0.87
15q25.3	rs8041189	15:85686327	G/A	0.70	intergenic	*PDE8A*	0.022	0.004	6.44	+−??	9,486/39,315	**7.30** **×** **10**^**−11**^	**1.24** **×** **10**^**−10**^	0.31
3p25	rs4685022	3:13832611	G/A	0.65	intergenic	*WNT7A*	0.019	0.004	6.40	+++?	9,576/39,654	**2.36** **×** **10**^**−09**^	**1.58** **×** **10**^**−10**^	0.11
2p16.1	rs7596872	2:56128091	C/A	0.90	intronic	*EFEMP1*	0.033	0.006	6.31	+−??	9,333/38,442	**1.00** **×** **10**^**−10**^	**2.80** **×** **10**^**−10**^	0.11
17q21.31	rs1126642	17:42989063	C/T	0.96	exonic	*GFAP*	0.051	0.009	6.23	+?+?	9,119/37,466	**6.19** **×** **10**^**−10**^	**4.67** **×** **10**^**−10**^	0.72
3q29	rs687610^d^	3:193515781	G/C	0.43	intergenic	*OPA1*	0.021	0.004	6.20	+++−	9,614/39,822	**2.99** **×** **10**^**−10**^	**5.81** **×** **10**^**−10**^	0.76
6p25.2	rs4959689	6:2617122	C/A	0.58	intergenic	*C6orf195*	0.020	0.004	6.03	++++	9,582/39,695	**3.37** **×** **10**^**−09**^	**1.63** **×** **10**^**−09**^	1.00
20q13.12	rs56104388	20:45302135	T/C	0.99	intronic	*SLC13A3*	0.101	0.017	5.85	+???	7,626/30,916	**4.80** **×** **10**^**−09**^	**4.80** **×** **10**^**−09**^	1.00
11q13.3	rs12417836	11:70089700	T/C	0.07	intergenic	*FADD*, *PPFIA1*	0.034	0.007	5.58	+−+?	9,464/38,960	**1.56** **×** **10**^**−08**^	**2.47** **×** **10**^**−08**^	0.40
8p11.21	rs2923437^d^	8:42425399	A/C	0.41	intergenic	*SMIM19*, *CHRNB3*, *SLC20A2*	0.018	0.004	5.49	++−−	9,614/39,822	**4.73** **×** **10**^**−08**^	**4.08** **×** **10**^**−08**^	0.14
6p25.3	rs1922930	6:1364691	C/A	0.12	intergenic	*FOXQ1*, *FOXF2*	0.029	0.006	5.47	++??	9,406/38,748	**3.60** **×** **10**^**−08**^	**4.62** **×** **10**^**−08**^	0.48
19p13.11	rs2385089	19:18550434	A/C	0.74	intergenic	*ISYNA1*, *ELL*, *LRRC25*^c^	0.023	0.005	5.49	+++−	9,614/39,822	**4.14** **×** **10**^**−08**^	5.73 × 10^−08^	0.57
7q33	rs10954468	7:134434661	C/A	0.40	intergenic	*BPGM*, *CALD1*^c^	0.019	0.004	5.52	+−?+	9,524/39,483	**3.39** **×** **10**^**−08**^	8.79 × 10^−08^	0.29
**PVS in basal ganglia (BG-PVS)**														
2q33.2	rs4675310^d^	2:203880834	A/G	0.87	intronic	*NBEAL1*, *ICA1L*	0.027	0.005	5.92	++??	9,011/39,243	**2.71** **×** **10**^**−09**^	**3.27** **×** **10**^**−09**^	0.64
3q26.31	rs6769442	3:171565463	G/A	0.75	intronic	*TMEM212*	0.020	0.004	5.74	++?+	9,101/39,788	**1.68** **×** **10**^**−08**^	**9.34** **×** **10**^**−09**^	0.96
**PVS in hippocampus (HIP-PVS)**														
1q25.3	rs10797812^d^	1:182984597	A/G	0.54	intergenic	*SHCBP1L*, *LAMC1*	0.027	0.004	7.84	++++	9,399/40,095	**1.67** **×** **10**^**−14**^	**4.39** **×** **10**^**−15**^	0.68
2p16.1	rs78857879^d^	2:56135099	G/A	0.90	intronic	*EFEMP1*	0.038	0.006	6.43	+???	9,033/38,008	**8.20** **×** **10**^**−11**^	**1.31** **×** **10**^**−10**^	1.00
1q41	rs6540873	1:215137222	A/C	0.62	intergenic	*CENPF*, *KCNK2*	0.020	0.004	5.95	+−−+	9,399/40,095	**1.38** **×** **10**^**−09**^	**2.72** **×** **10**^**−09**^	0.11

EA, effect allele; OA, other allele; EAF, effect allele frequency; *Z*-scores of the sample size-weighted cross-ancestry meta-analysis are represented, except for the two SNPs reaching genome-wide significance in Europeans only (rs2385089, rs10954468) for which the European meta-analysis *Z*-score is reported; dir, the association direction of the EA with the phenotype (extensive PVS burden versus the rest) for European, Hispanic, Asian and African-American ancestry studies, in this order; *N* ext-PVS, the number of participants with extensive PVS burden in the cross-ancestry meta-analysis, in each location (WM-PVS, BG-PVS, HIP-PVS); *N* total, the total number of participants in the cross-ancestry meta-analysis; *P* value EUR, *P* value in the European meta-analysis; *P* value All, *P* value in the cross-ancestry meta-analysis; Het *P* value corresponds to the heterogeneity *P* value in the meta-analysis (except for rs2385089 and rs10954468 for which the European meta-analysis Het *P* value is reported); *P* values for genome-wide significant loci (*P* < 5 × 10^−8^) are in bold; PVS GWAS analyses in individual cohorts were adjusted for age, sex and intracranial volume (or brain parenchymal fraction for ASPS), principal components of population stratification, and study site.

^a^From cross-ancestry inverse variance-weighted meta-analysis.

^b^From cross-ancestry *Z*-score-based meta-analysis.

^c^Genome-wide significant association in Europeans only.

^d^For these loci, the lead SNP was different in the European meta-analysis (Cross-ancestry lead SNP → European lead SNP: rs72485816→rs6094423; rs687610→rs6444747; rs2923437→rs62509329; rs4675310→rs140244541; rs10797812→rs2022392; rs78857879→rs7596872); the *P* value corresponding to the European lead SNP is reported under "*P* value EUR" (*r*² > 0.50 between the European and cross-ancestry lead SNPs at these loci).

We performed conditional logistic regression using Genome-wide Complex Trait Analysis (GCTA)-COJO (Methods) to seek independent association signals within genome-wide significant loci. Consistent with linkage disequilibrium (LD)-based clumping, this identified two independent signals at chr3p25.1 (*WNT7A*) and six at chr20q13.12 (*SLC13A3*; Supplementary Fig. 1 and Supplementary Table 5a), four of which with low-frequency variants (Table 1). The six polymorphisms at chr20q13.12 generated eight haplotypes with haplotypic *R*^2^ (percentage of haplotypic variability explained by observed genotypes) > 0.7 in the Three-City Dijon Study (3C-Dijon) cohort, of European ancestry (*N* = 1,500; Supplementary Table 5b). The two common rs2425881-A and rs2425884-C alleles, in very low LD with each other (*r*^2^ (a measure of correlation of alleles for two genetic variants) ~ 0.05, *D*′ (a pairwise *r*^2^ standardized for allele frequencies) ~ 0.50), generated a common haplotype that was more frequent in individuals with extensive WM-PVS than in those without (0.50 versus 0.47, odds ratio (OR) = 1.19 (95% confidence interval (95% CI), 0.99–1.43)). The effect of this haplotype was amplified by 1.7 in the presence of the rs112407396-T allele (MAF = 0.03), which has a high probability of being a regulatory variant (HaploReg, GTex, RegulomeDB). Next, to account for allelic heterogeneity between ancestries, we conducted cross-ancestry meta-analyses with MR-MEGA (Methods). There were no loci showing high heterogeneity in allelic effects across ancestries (*P*_Het_ < 0.01) and reaching genome-wide significance (Supplementary Table 6).

Using MAGMA and VEGAS, we performed gene-based association analyses in participants of European ancestry, testing the combined association of variants within a gene with PVS (Methods). MAGMA identified 28 gene-wide significant associations (*P* < 2.63 × 10^−6^), of which 12 in 8 loci did not reach genome-wide significance in the GWAS (WM-PVS: 3 (*INS-IGF2/IGF2, PRKAG2, LRP4/CKAP5*); BG-PVS: 4 (*SH3PXD2A, WNT3, ZMYND15*, *KCNRG/TRIM13/SPRYD7*); and HIP-PVS: 1 (*PDZRN4*); Fig. 1 and Supplementary Table 7). VEGAS identified one additional gene (*NSF*) for BG-PVS (same locus as *WNT3*; Supplementary Table 7). All were in suggestive GWAS loci (*P* < 5 × 10^−6^; Supplementary Table 8).

Using LD-score regression, we estimated heritability at 11% for WM-PVS, 5% for BG-PVS and 8% for HIP-PVS (Methods and Supplementary Table 9). We found moderate genetic correlation between BG-PVS and HIP-PVS (*r*_g_ (SE) = 0.63 (0.14), *P* = 7.23 × 10^−6^), and modest genetic correlation of WM-PVS with BG-PVS (*r*_g_ (SE) = 0.24 (0.12), *P* = 0.055) and HIP-PVS (*r*_g_ (SE) = 0.27 (0.09), *P* = 0.003). The genetic correlation of PVS in CHARGE and UKB was moderate to high for WM-PVS and HIP-PVS and weaker for BG-PVS (Supplementary Table 10). Associations with genome-wide significant PVS loci were highly consistent between the UKB and CHARGE contributions and between the two dichotomous and the continuous PVS measures in UKB (Methods and Supplementary Tables 11 and 12). In sensitivity analyses in two representative cohorts (UKB and 3C-Dijon), continuous and dichotomous PVS measures were strongly correlated (Spearman’s *ρ*, 0.61–0.80; Supplementary Table 13).

To increase statistical power, we conducted secondary multivariate association analyses using Multi-Trait Analysis of GWAS (MTAG) (Methods), including summary statistics from GWAS of other cSVD markers (WMH volume, lacunes; Supplementary Table 14). We observed the highest gain in power for BG-PVS: ten additional loci reached genome-wide significance, of which two also for HIP-PVS (*STN1*, *DEGS2/EVL*). Two MTAG BG-PVS loci (*CACNB2*, *NSF/WNT3*) and one MTAG WM-PVS locus (*VWA2*) were not described before with any MRI marker of cSVD. Six loci showed greater significance in MTAG than with PVS, WMH volume or lacunes alone: at *VWA2* (WM-PVS); *SH3PXD2A/STN1*, *COL4A2*, *CACNB2* and *NSF/WNT3* (BG-PVS); and *DEGS2/EVL* (BG-PVS, HIP-PVS).

Using WES data and exome content of WGS data in 19,010 participants from UKB and the Brain Imaging, cognition, dementia, and next-generation genomics (BRIDGET) consortium (Methods and Supplementary Table 1), of whom 4,531, 4,424 and 4,497 had extensive PVS in WM, BG and hippocampus, we identified 19 variants in the chr1q25.3 locus associated with HIP-PVS, including two missense variants (rs20563 and rs20558) and one splice donor insertion (rs34133998) in *LAMC1* at *P* < 5 × 10^−8^, in strong LD with the GWAS sentinel variant (Supplementary Table 15a). Gene-based burden tests exploring protein-modifying rare variants (MAF < 0.01) did not identify any gene-wide significant association (Supplementary Table 15b).

### Follow-up of findings across the lifespan and ancestries

We explored associations of WM-PVS and BG-PVS risk variants with these phenotypes in young adults (Internet-based Students’ HeAlth Research Enterprise (i-Share) study, *N* = 1,748, 22.1 ± 2.3 yr) and in older Japanese community-dwelling people (Nagahama study, *N* = 2,862, 68.3 ± 5.3 yr; Methods). We used an artificial intelligence-based method to derive quantitative WM-PVS and BG-PVS burden (HIP-PVS not available) and dichotomized it (top quartile versus the rest; Supplementary Table 2). In total, 67% of WM-PVS loci reached nominally significant associations in at least one of the two follow-up cohorts (*P* < 0.05 in i-Share and/or Nagahama), 43% of which at *P* < 1.09 × 10^−3^ (correcting for the number of loci tested), with consistent directionality of effect (a binomial test showed significant concordance of risk alleles; Supplementary Table 12b). In i-Share, 52% of WM-PVS risk variants were associated with WM-PVS (*P* < 0.05, of which 4 at *P* < 1.09 × 10^−3^; Table 2 and Supplementary Table 12a). A WM-PVS rescaled weighted genetic risk score (rwGRS) derived from European GWAS loci was associated with WM-PVS in i-Share (OR = 1.16 (95% CI, 1.08–1.24), *P* = 5.89 × 10^−6^ and β (SE) = 0.064 (0.007), *P* = 2.06 × 10^−19^ for dichotomous and continuous measures; Supplementary Fig. 2). Although meta-regression suggested larger effect sizes at younger ages for lead variants at *OPA1* and S*LC13A3*, differences were not significant after removing the much younger i-Share cohort (Supplementary Fig. 3). In Nagahama, out of 15 available WM-PVS risk loci (six were rare or monomorphic), eight loci (53%) were associated with continuous PVS burden at *P* < 0.05, of which four at *P* < 1.09 × 10^−3^ and one at genome-wide significance (at *SLC13A3*; Table 2 and Supplementary Table 12a). A European WM-PVS weighted genetic risk score (wGRS) combining 14 independent loci (1000 Genomes project (1000G) Japanese reference panel) was associated with WM-PVS in Nagahama (OR = 1.18 (95% CI, 1.13–1.24), *P* = 5.68 × 10^−13^ and β (SE) = 0.01 (0.001), *P* = 7.18 × 10^−18^ for dichotomous and continuous measures). Although HIP-PVS data were not available in the follow-up cohorts, two of the three HIP-PVS loci were shared with WM-PVS and replicated with that phenotype.

**Table 2:** Association of genome-wide significant WM- and BG-PVS risk loci with PVS burden across the lifespan (i-Share study, *N* = 1,748) and across ancestries (Nagahama study, *N* = 2,862)

GWAS meta-analysis				i-Share (dichotomous)		i-Share (continuous)		Nagahama (dichotomous)		Nagahama (continuous)	
SNP	chr:position	EA/OA	Nearest gene(s)	OR (95% CI)	*P* value	β (SE)	*P* value	OR (95% CI)	*P* value	β (SE)	*P* value
**PVS in white matter (WM-PVS)**											
rs6011998	20:45269867	C/T	*SLC13A3*	1.26 (0.83–1.92)	0.28	0.164 (0.04)	**4.20** **×** **10**^**−05**^^a^	1.69 (1.33–2.13)	**1.22** **×** **10**^**−05**^^a^	0.037 (0.008)	**6.21** **×** **10**^**−07**^^a^
rs13079464	3:13822439	C/G	*WNT7A*	1.12 (0.91–1.40)	0.29	0.014 (0.02)	0.50	1.16 (0.97–1.40)	0.11	0.015 (0.006)	**1.50** **×** **10**^**−02**^
rs2425884	20:45258292	C/T	*SLC13A3*	1.18 (0.95–1.45)	0.13	0.077 (0.02)	**2.98** **×** **10**^**−**^^**04**a^	1.29 (1.09–1.52)	**3.48** **×** **10**^**−03**^	0.026 (0.005)	**1.77** **×** **10**^**−06**^^a^
rs10817108	9:113658671	A/G	*LPAR1*	0.90 (0.69–1.17)	0.44	0.058 (0.03)	**2.23** **×** **10**^**−**^^**02**^	1.18 (0.98–1.43)	0.07	0.017 (0.006)	**4.10** **×** **10**^**−03**^
rs2425881	20:45255618	A/G	*SLC13A3*	1.47 (1.03–2.01)	**1.40** **×** **10**^**−02**^	0.063 (0.03)	**2.62** **×** **10**^**−02**^	1.18 (1.01–1.37)	**3.66** **×** **10**^**−02**^	0.014 (0.005)	**4.68** **×** **10**^**−03**^
rs3772833	3:124518362	G/A	*ITGB5*, *UMPS*	1.22 (0.89–1.66)	0.21	0.006 (0.03)	0.85	1.06 (0.88–1.29)	0.51	0.008 (0.006)	0.16
rs112407396	20:45276381	T/A	*SLC13A3*	1.47 (0.77–2.78)	0.24	0.147 (0.07)	**3.13** **×** **10**^**−02**^	NA	NA	NA	NA
rs10494988	1:215141570	C/T	*CENPF*, *KCNK2*	1.18 (0.95–1.47)	0.14	0.079 (0.02)	**1.94** **×** **10**^**−04**^^a^	1.01 (0.86–1.18)	0.90	−0.002 (0.005)	0.67
rs72485816	20:45314435	T/C	*TP53RK*, *SLC13A3*	1.01 (0.56–1.80)	0.98	0.093 (0.06)	0.095	1.32 (1.10–1.59)	**2.83** **×** **10**^**−03**^	0.033 (0.006)	**1.91** **×** **10**^**−08**^^b^
rs8041189	15:85686327	G/A	*PDE8A*	1.14 (0.89–1.44)	0.30	0.041 (0.02)	0.073	1.67 (0.84–3.33)	0.14	0.046 (0.021)	**2.40** **×** **10**^**−02**^
rs4685022	3:13832611	G/A	*WNT7A*	1.12 (0.88–1.42)	0.34	0.023 (0.02)	0.31	1.15 (0.97–1.36)	0.10	0.010 (0.005)	0.075^c^
rs7596872	2:56128091	C/A	*EFEMP1*	1.65 (1.10–2.46)	**1.14** **×** **10**^**−02**^	**0.089 (0.03)**	**1.10** **×** **10**^**−02**^	NA	NA	NA	NA
rs1126642	17:42989063	C/T	*GFAP*	1.13 (0.65–1.97)	0.67	0.127 (0.05)	**1.27** **×** **10**^**−02**^	1.35 (1.09–1.67)	0.11	0.033 (0.007)	**9.88** **×** **10**^**−07**^^a^
rs687610	3:193515781	G/C	*OPA1*	1.46 (1.18–1.80)	**4.88** **×** **10**^**−04**^^a^	**0.109 (0.02)**	**1.29** **×** **10**^**−07**^^a^	0.95 (0.81–1.13)	0.59	0.006 (0.005)	0.28
rs4959689	6:2617122	C/A	*C6orf195*	1.10 (0.89–1.37)	0.37	0.022 (0.02)	0.30	NA	NA	0.024 (0.026)	0.34^c^
rs56104388	20:45302135	T/C	*SLC13A3*	1.30 (0.40–4.24)	0.67	0.274 (0.11)	**1.47** **×** **10**^**−02**^	NA	NA	NA	NA
rs12417836	11:70089700	T/C	*FADD*, *PPFIA1*	0.99 (0.64–1.56)	0.99	0.045 (0.04)	0.29	0.87 (0.62–1.21)	0.40	−0.002 (0.011)	0.84
rs2923437	8:42425399	A/C	*SMIM19*, *CHRNB3*, *SLC20A2*	0.98 (0.78–1.23)	0.88	0.047 (0.02)	**2.60** **×** **10**^**−02**^	1.11 (0.94–1.31)	0.23	0.008 (0.005)	0.11
rs1922930	6:1364691	C/A	*FOXQ1*, *FOXF2*	0.93 (0.65–1.33)	0.70	0.035 (0.04)	0.33	NA	NA	NA	NA
rs2385089	19:18550434	A/C	*ISYNA1*, *ELL*, *LRRC25*	1.20 (0.94–1.53)	0.14	0.049 (0.03)	0.057	NA	NA	NA	NA
rs10954468	7:134434661	C/A	*BPGM*, *CALD1*	1.08 (0.86–1.36)	0.50	0.033 (0.02)	0.13	NA	NA	NA	NA
**PVS in basal ganglia (BG-PVS)**											
rs4675310^d^	2:203880834	A/G	*NBEAL1*, *ICA1L*	1.07 (0.81–1.41)	0.61	0.01 (0.04)	0.78	1.88 (0.63-5.60)	0.26	0.046 (0.03)	0.11
rs6769442	3:171565463	G/A	*TMEM212*	1.10 (0.87–1.40)	0.37	0.03 (0.03)	0.33	1.04 (0.71-1.53)	0.82	0.008 (0.01)	0.35

NA in the Nagahama Study correspond to variants that are rare (MAF < 1%: rs7596872; rs1922930; rs10954468) or monomorphic (rs112407396; rs56104388) in East Asians, or not available including in EAS 1000G data (rs2385089).

Analyses were adjusted for age, sex and intracranial volume, principal components of population stratification in the i-Share and Nagahama studies, and additionally adjusted for study center in the Nagahama study. In the Nagahama study, when the lead SNP from the PVS GWAS meta-analysis was not present, we used a tag SNP with *r*^2^ > 0.80 using the 1000G Japanese reference panel.

SNPs or tag SNPs (*r*² > 0.80, 1000G EAS) with a *P* < 0.05 are in bold.

^a^SNPs with a *P* < 1.09 × 10^−3^ (Bonferroni correction for 23 independent loci and two PVS locations).

^b^SNPs reaching genome-wide significance.

^c^The tag SNP (*r*² > 0.80) is nominally significant: rs4685022 (*r*² = 0.81 with rs934448, 1000G EAS), *P* = 0.048; rs4959689 (*r*² = 0.83 with rs1772953, 1000G EAS), *P* = 0.02.

^d^The lead SNP for this locus is not present in the Nagahama study; we used a tag SNP (rs150788469, *r*² = 1.0 with rs4675310) where the A allele of rs4675310 is in phase with the G allele of rs150788469.

### Clinical correlates of identified PVS loci

**Fig. 2:**
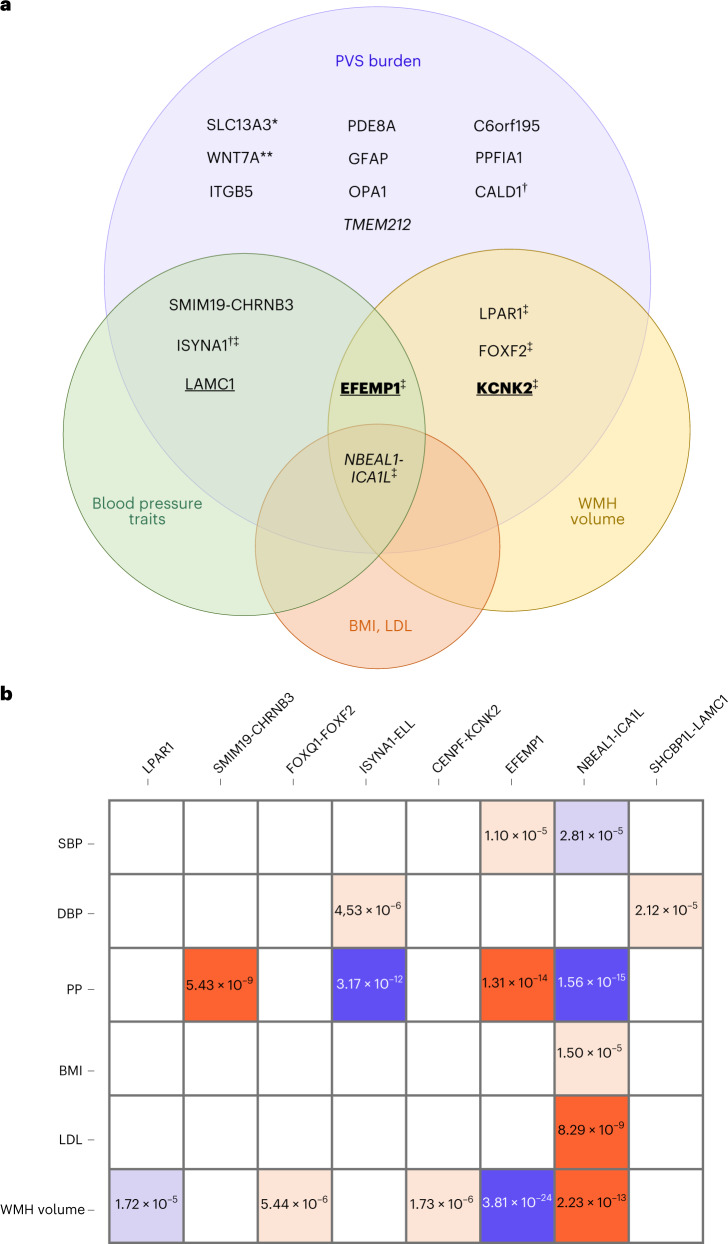
Association of PVS loci with vascular risk factors and other MRI markers of cSVD. **a**, Venn diagram displaying significant association of genome-wide significant risk loci for PVS burden with vascular risk factors and other MRI markers of cSVD: in italics for BG-PVS; underlined for HIP-PVS; underlined and in bold for HIP- and WM-PVS; all others for WM-PVS only (*P* < 3.3 × 10^−5^, two-sided, correcting for multiple testing (21 independent phenotypes, 3 PVS locations and 24 independent loci)); *6 independent loci; **2 independent loci; ^†^genome-wide significant in Europeans only; ^‡^in colocalization analyses the posterior probability PP4 was higher than 75% for these loci (only with WMH at *NBEAL1-ICA1L*). Exact *P* values are provided in Supplementary Table 16. **b**, Direction of association and level of significance of pleiotropic SNPs displayed in **a**: in red when the risk allele for extensive PVS burden is positively associated with the trait, in blue when the PVS risk allele is negatively associated with the trait (unexpected direction), in dark red and dark blue for genome-wide significant associations and in light red and light blue for significant association after multiple-testing correction (*P* < 3.3 × 10^−5^, two-sided, correcting for multiple testing (21 independent phenotypes, 3 PVS locations and 24 independent loci)). PP, pulse pressure; BMI, body mass index; LDL, LDL cholesterol.

We examined whether PVS risk loci (lead and proxy variants with *r*^2^ > 0.9) were associated with MRI markers of brain aging, putative risk factors (vascular risk factors and sleep patterns) and common neurological diseases (stroke, Alzheimer’s disease, Parkinson’s disease), using the largest published GWAS (Methods). Of 24 independent PVS risk loci, five (21%) were significantly (*P* < 3.3 × 10^−5^) associated with WMH volume and five (21%) with blood pressure traits (in the same and opposite directions; Fig. 2). Colocalization analyses suggested a shared causal variant for two-thirds of these associations (posterior probability for a shared causal variant, PP4 > 0.75; Supplementary Table 16). Sixteen PVS loci (67%) did not show any association with vascular or neurological traits, thus pointing to pathways that do not seem mediated by established risk factors (Methods and Supplementary Tables 16 and 17).

**Fig. 3:**
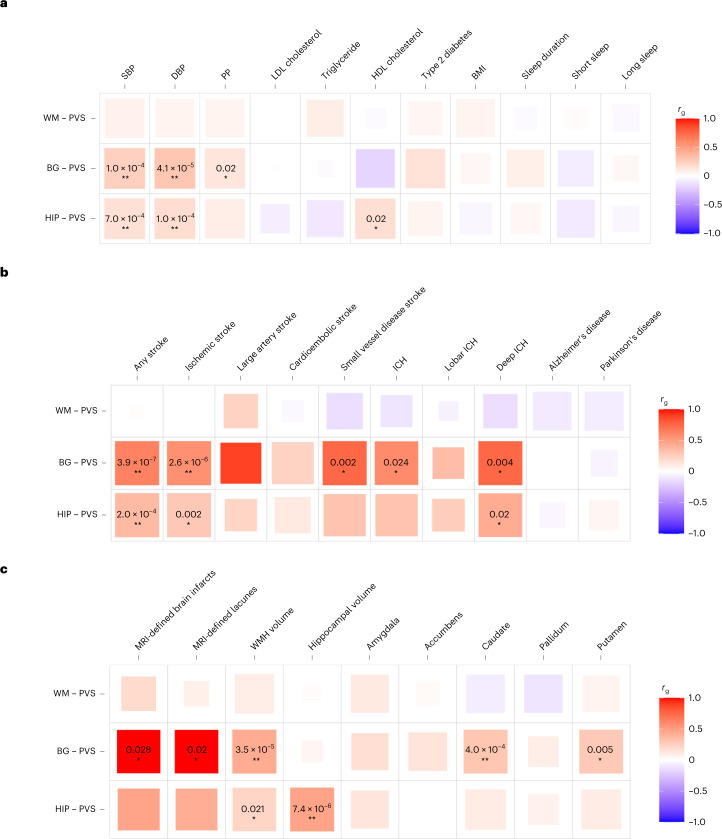
Genetic correlations of extensive PVS burden with risk factors, neurological diseases and other MRI markers of brain aging. **a**–**c**, Genetic correlation using LD-score regression of extensive PVS burden with putative risk factors (**a**), neurological diseases (**b**) and other MRI markers of brain aging (**c**); two-sided exact *P* values are provided for nominally significant results (*P < 0.05) and significant results after multiple-testing correction (**P < 7.9 × 10^−4^, correcting for 21 independent phenotypes and the three PVS locations); full results are provided in Supplementary Table 9. Larger colored squares correspond to more significant *P* values and the colors represent the direction of the genetic correlation (positive in red, negative in blue). HDL, high-density lipoprotein; amygdala, accumbens (nucleus), caudate (nucleus), pallidum, and putamen correspond to the volumes of these subcortical structures.

Second, we explored genetic correlations of PVS burden with the same traits using LD-score regression (Methods, Fig. 3 and Supplementary Table 9). We observed significant (*P* < 7.9 × 10^−4^) genetic correlation of BG-PVS with larger WMH and caudate nucleus volumes, and of HIP-PVS with larger hippocampal volume. BG-PVS and HIP-PVS showed significant genetic correlation with higher systolic blood pressure (SBP), diastolic blood pressure (DBP), any stroke and ischemic stroke, and nominally significant genetic correlation with (deep) intracerebral hemorrhage (ICH). Genetic correlations were consistent in secondary analyses conducted separately in CHARGE and UKB (Supplementary Table 9).

**Extended Data Fig. 2:**
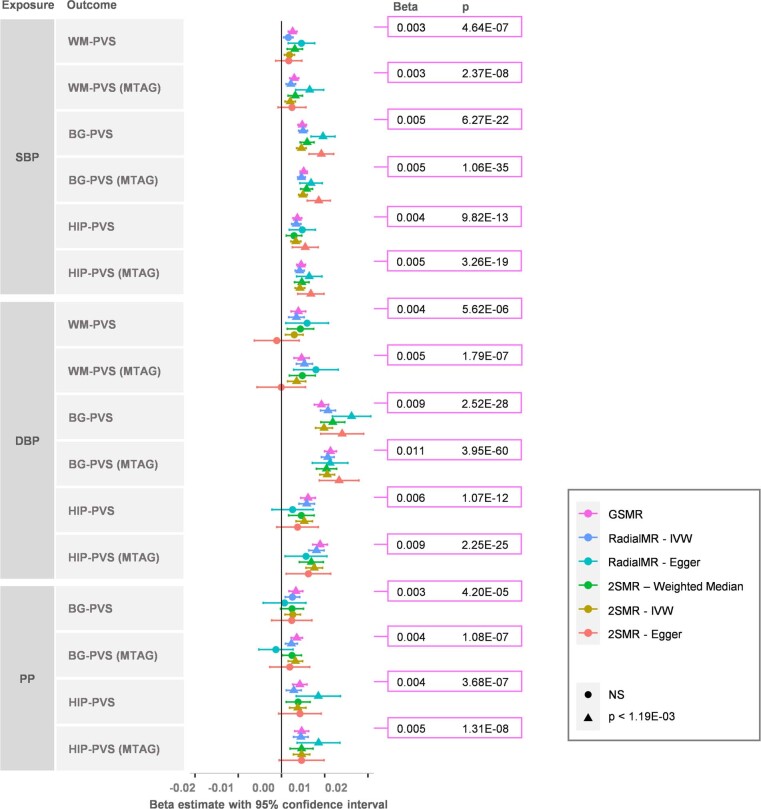
Two-sample Mendelian randomization analysis between putative risk factors and extensive PVS burden. Two-sample Mendelian randomization was conducted using European PVS GWAS summary statistics (N = 38,598 (WM-PVS), N = 38,903 (BG-PVS) and N = 38,871 (HIP-PVS)), combined with summary statistics for lacunes (N = 1,715 cases / N = 15,096 controls) and WMH volume (N = 48,454) in MTAG, for the outcomes and European GWAS summary statistics for blood pressure traits (N = 757,601) to generate instruments for exposures (Supplementary Tables 1 and 29). Only significant associations after multiple testing correction (p < 1.19×10^−3^) in GSMR are shown. Each dot (or triangle if p < 1.19×10^−3^) represents the beta estimate from Mendelian randomization with lines representing the 95% confidence interval. Two-sided p-values of GSMR are reported. PVS indicates perivascular spaces; WM, white matter; BG, basal ganglia; HIP, hippocampus; MTAG, multi-trait analysis of genome-wide association summary statistics; DBP, diastolic blood pressure; PP, pulse pressure; SBP, systolic blood pressure; NS, not significant; GSMR, Generalised Summary-data-based Mendelian Randomisation; IVW, Inverse variance weighted; 2SMR, Two-SampleMR.

**Extended Data Fig. 3:**
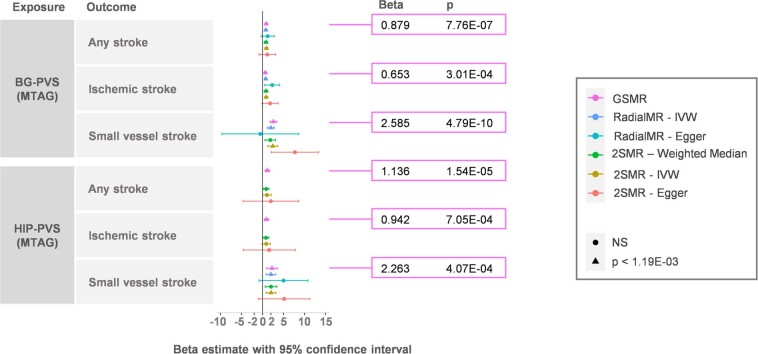
Two-sample Mendelian randomization analysis between extensive PVS burden and neurological traits. Two-sample Mendelian randomization was conducted using European GWAS summary statistics for any stroke (N = 40,585 cases / N = 406,111 controls), ischemic stroke (N = 34,217 cases / N = 400,201 controls) and small vessel stroke (N = 5,386 cases / N = 254,558 controls) for outcomes and European PVS GWAS summary statistics (N = 38,598 (WM-PVS), N = 38,903 (BG-PVS) and N = 38,871 (HIP-PVS)), combined with summary statistics for lacunes (N = 1,715 cases / N = 15,096 controls) and WMH volume (N = 48,454) in MTAG, to generate instruments for exposures (Supplementary Tables 1 and 29). Only significant associations after multiple testing correction (p < 1.19×10^−3^) in GSMR are shown. Each dot (or triangle if p < 1.19×10^−3^) represents the beta estimate from Mendelian randomization with lines representing the 95% confidence interval. Two-sided p-values of GSMR are reported. PVS indicates perivascular spaces; WM, white matter; BG, basal ganglia; HIP, hippocampus; MTAG, multi-trait analysis of genome-wide association summary statistics; NS, not significant; GSMR, Generalised Summary-data-based Mendelian Randomisation; IVW, Inverse variance weighted; 2SMR, Two-SampleMR.

Third, we used two-sample Mendelian randomization (MR) to seek evidence for a causal association of putative risk factors with PVS burden and of PVS burden with neurological diseases, using generalized summary-data-based MR (GSMR), and confirming significant associations (*P* < 1.19 × 10^−3^) with RadialMR, TwoSampleMR and MR-CAUSE (Methods). Genetically determined higher SBP and DBP were consistently associated with BG-PVS, HIP-PVS and WM-PVS, although for WM-PVS the association with SBP was only nominally significant in RadialMR (Supplementary Table 18 and Extended Data Fig. 2). There was no evidence for reverse causation using MR-Steiger, but some evidence of residual pleiotropy after removal of outlier variants for SBP and DBP (RadialMR), with significant evidence for a causal model in MR-CAUSE for BG-PVS. Genetic liability to BG-PVS and HIP-PVS derived from a multi-trait analysis accounting for other MRI markers of cSVD (MTAG) was associated with an increased risk of any stroke, ischemic stroke and small vessel stroke (SVS) for BG-PVS, and SVS for HIP-PVS, suggesting that shared pathways between PVS, WMH and lacunes may be causally associated with stroke (Supplementary Table 18 and Extended Data Fig. 3). In multivariable MR analyses accounting for SBP and DBP, genetic liability to BG-PVS and HIP-PVS was significantly associated with an increased risk of any stroke, ischemic stroke and SVS (Supplementary Table 19).

### Functional exploration of identified PVS loci

Using MAGMA and VEGAS2Pathway (Methods), we identified significant enrichment of PVS loci in pathways involved in extracellular matrix (ECM) structure and function, lymphatic endothelial cell differentiation, cell motility and thyroid hormone transport (Supplementary Tables 20 and 21).

**Extended Data Fig. 4:**
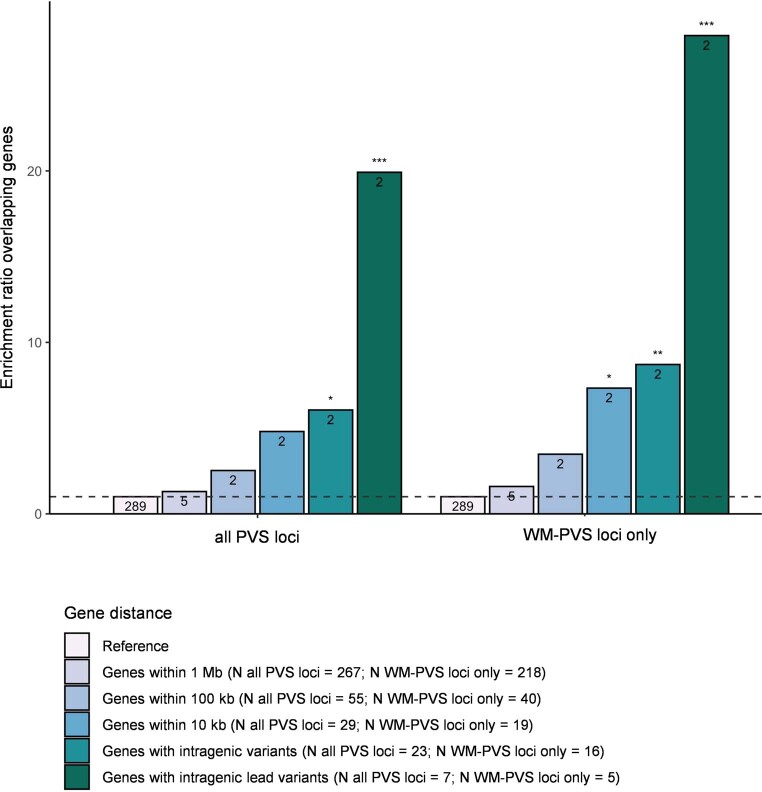
Enrichment of PVS risk loci in genes mutated in OMIM syndromes. Enrichment of all perivascular spaces (PVS) loci (left) and WM-PVS loci only (right) in genes mutated in OMIM syndromes associated with white matter hyperintensities, leukodystrophy, leukoencephalopathy, according to distance from the lead variant; * p < 0.05; ** p < (0.05/5); *** p < (0.05/5/2). Exact p-values for all PVS loci: p = 0.041 (genes with intragenic SNPs), p = 9,27×10^−06^ (genes with intragenic lead SNPs); exact p-values for WM-PVS loci only: p = 0,018 (genes within 10 kb distance), p = 0.008 (genes with intragenic SNPs), p = 8,61×10^−08^ (genes with intragenic lead SNPs), full results are provided in Supplementary Table 22.

Genes closest to PVS lead risk variants were significantly enriched in genes mutated in Online Mendelian Inheritance in Man (OMIM) syndromes associated with leukodystrophy, leukoencephalopathy or WMH, with a 20-fold enrichment in genes containing an intragenic lead variant. This enrichment was 30-fold when focusing on WM-PVS loci only, comprising several genes involved in early-onset leukodystrophies: *GFAP* (chr17q21.31), mutations of which cause Alexander disease, a rare neurodegenerative disorder of astrocytes leading to psychomotor regression and death; *SLC13A3* (chr20q13.12), causing acute reversible leukoencephalopathy with increased urinary alpha-ketoglutarate; and *PNPT1* (chr2p16.1), causing Aicardi–Goutières syndrome and cystic leukoencephalopathy (Methods, Extended Data Fig. 4 and Supplementary Table 22). Although several genes near PVS lead risk variants were described to be involved in glioma, we found no significant enrichment for glioma genes (Methods).

**Fig. 4:**
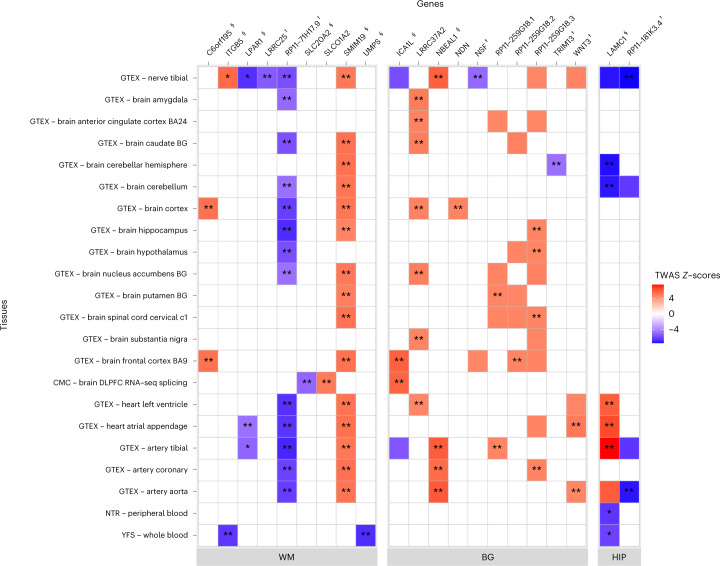
Transcriptome-wide significant genes with extensive PVS burden. We used precomputed functional weights from 22 publicly available gene expression reference panels from brain (GTEx v7, CommonMind Consortium (CMC)), peripheral nerve tissues (GTEx v7), heart and arteries (GTEx v7), and blood (Netherlands Twin Registry (NTR) and Young Finns Study (YFS)). Transcriptome-wide significant genes (eGenes) and the corresponding eQTLs were determined using Bonferroni correction, based on the average number of features (4,235 genes) tested across all tissues and correcting for the three independent PVS locations (*P* < 3.93 × 10^−6^). *Significant result in the TWAS and conditional analyses; **significant result in the TWAS and conditional analyses, and with a COLOC PP4 > 0.75; eGenes for loci identified in the GWAS (^†^), gene-based test (^‡^) or both GWAS and gene-based test (^§^).

To seek evidence for a causal implication of specific genes and variants, we performed transcriptome-wide association studies (TWAS) using TWAS-Fusion (Methods), with European PVS GWAS summary statistics and the GTEx v7 multi-tissue (RNA sequencing) database, focusing on brain, vascular and blood tissues. We found 36 transcriptome-wide significant expression–trait associations for WM-PVS, 25 for BG-PVS and seven for HIP-PVS that were significant in colocalization analyses (TWAS-COLOC), providing evidence of a shared causal variant between the corresponding gene expression and PVS (Supplementary Table 23). Most genes with significant expression–trait associations (12) were in genome-wide significant PVS risk loci: eight genes in five WM-PVS GWAS loci (*C6orf195*, *ITGB5*, *LPAR1*, *LRRC25*, *RP11-71H17.9*, *SLC20A2*, *SMIM19*, *UMPS*), two genes in one BG-PVS GWAS locus (*ICA1L*, *NBEAL1*) and two genes in an HIP-PVS GWAS locus (*LAMC1* and *RP11-181K3.4*), while nine were outside GWAS loci, requiring confirmation (Fig. 4). TWAS-COLOC signals were mostly observed in brain tissues (17 genes), but also in vascular tissues (ten genes) and blood (two genes).

To identify enrichment in specific brain cell types, we used a recently developed pipeline combining three cell type enrichment methods, stratified LD-score, MAGMA and H-MAGMA (Supplementary Table 24). We observed significant enrichment in brain vascular endothelial cells for all PVS locations, based on a human single-cell atlas of fetal gene expression, and in pericytes and astrocytes for WM-PVS (Supplementary Tables 24 and 25).

**Extended Data Fig. 5:**
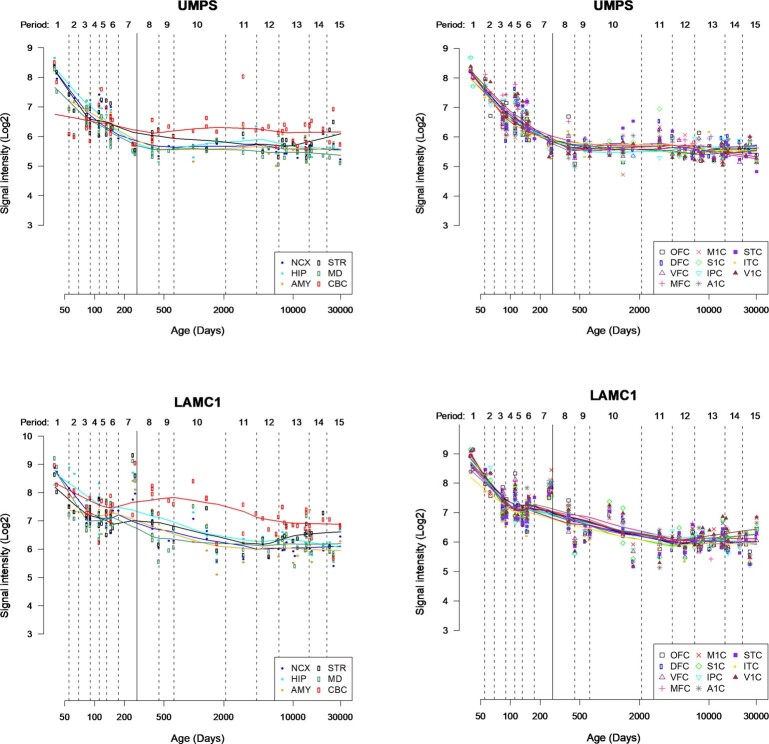
Brain expression pattern across the lifespan of genes near genome-wide significant PVS loci that are also identified in the TWAS and peaking in the pre-natal period (*UMPS* and *LAMC1*). The figure displays brain expression patterns across the lifespan of the 2 significant PVS TWAS-COLOC genes from genome-wide significant PVS GWAS loci peaking in the pre-natal period (*UMPS* for WM-PVS and *LAMC1* for HIP-PVS) (see Supplementary Table 4 for brain expression pattern across the lifespan of other genes). The spatio-temporal gene expression level is plotted as log2-transformed exon array signal intensity (y-axis) against the post conception days (x-axis) as provided by the Human Brain Transcriptome project database. Periods of human development and adulthood are indicated by vertical dashed lines: 4-8 post conception weeks [PCW] (period 1), 8-10 PCW (period 2), 10-13 PCW (period 3), 13-16 PCW (period 4), 16-19 PCW (period 5), 19-24 PCW (period 6), 24-38 PCW (period 7), birth-6 postnatal months (period 8), 6-12 postnatal months (period 9), 1-6 years (period 10), 6-12 years (period 11), 12-20 years (period 12), 20-40 years (period 13), 40-60 years (period 14), and 60 years + (period 15). The boundary between pre- and postnatal periods is indicated by the solid vertical line. Each colored point represents the expression level of each gene across 16 anatomical brain regions and ages. Brain structure includes 11 neocortical areas (NCX, blue), and 5 subcortical regions: hippocampus (HIP, cyan), amygdala (AMY, orange), striatum (STR, black), mediodorsal nucleus of thalamus (MD, dark green), and cerebellar cortex (CBC, red). Neocortical areas include orbital prefrontal cortex (OFC), dorsolateral prefrontal cortex (DFC), ventrolateral prefrontal cortex (VFC), medial prefrontal cortex (MFC), primary motor cortex (M1C), primary somatosensory cortex (S1C), posterior inferior parietal cortex (IPC), primary auditory cortex (A1C), posterior superior temporal cortex (STC), inferior temporal cortex (ITC), and primary visual cortex (V1C).

We explored brain expression patterns from development to adulthood of genes nearest to PVS loci, prioritizing TWAS-COLOC genes (Methods). Several genes showed important variations in expression levels throughout the life course, some peaking in the prenatal period (for example, *LAMC1*, *UMPS*), suggestive of developmental mechanisms (Extended Data Fig. 5 and Supplementary Fig. 4).

**Extended Data Fig. 6:**
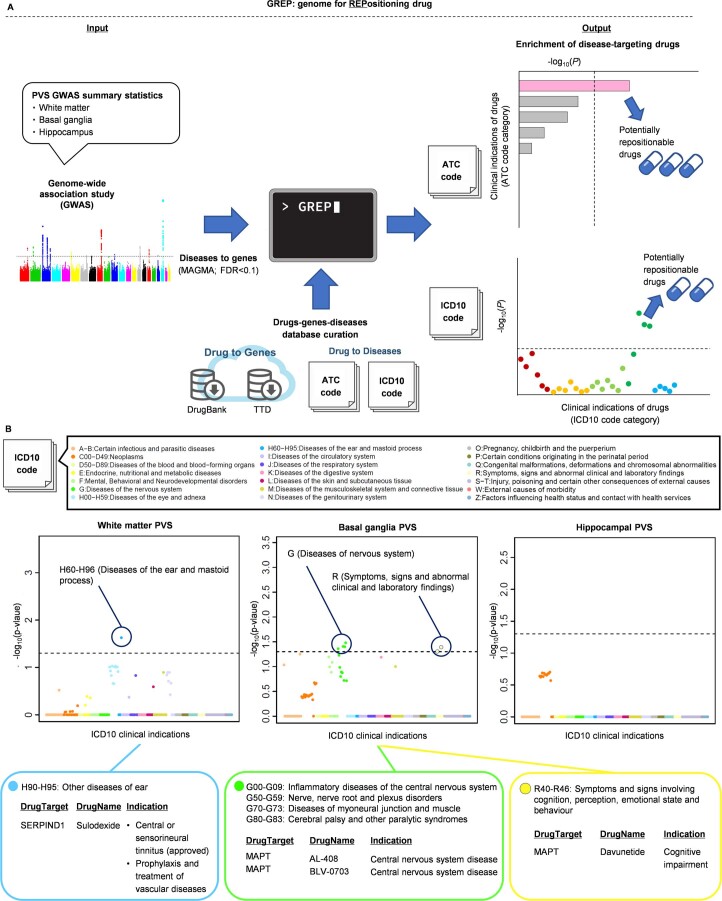
Enrichment of PVS genes in targets of drugs validated in other indications (Genome for REPositioning drugs, using ICD10 codes). Using the Genome for REPositioning drugs (GREP) software (**A**), we found a significant enrichment of BG-PVS genes in targets of drugs for diseases of the nervous system (ICD10 codes G50-G59, G70-G73, and G80-G83, OR = 51.0, 49.7, and 60.4, p = 3.90×10^−2^, 4.00×10^−2^, and 3.32×10^−2^) and for symptoms and signs involving cognition, perception, emotional state and behaviour (ICD10 codes R40-R46, OR = 48.4, p = 4.10×10^−2^), driven by MAPT (chr17q21.31), a target for davunetide (**B**). We also found a significant enrichment of HIP-PVS genes in targets of drugs for diseases of the ear (ICD10 codes H90-H95, OR = 57.6, p = 2.34×10^−2^), driven by SERPIND1 (chr22q11.21) a target for sulodexide, also used for the prophylaxis and treatment of vascular diseases with increased risk of thrombosis (**B**). PVS indicates perivascular spaces; ATC, Anatomical Therapeutic Chemical classification; ICD10, the 10^th^ revision of the International Statistical Classification of Diseases and Related Health Problems; TTD, Therapeutic Target Database.

**Extended Data Fig. 7:**
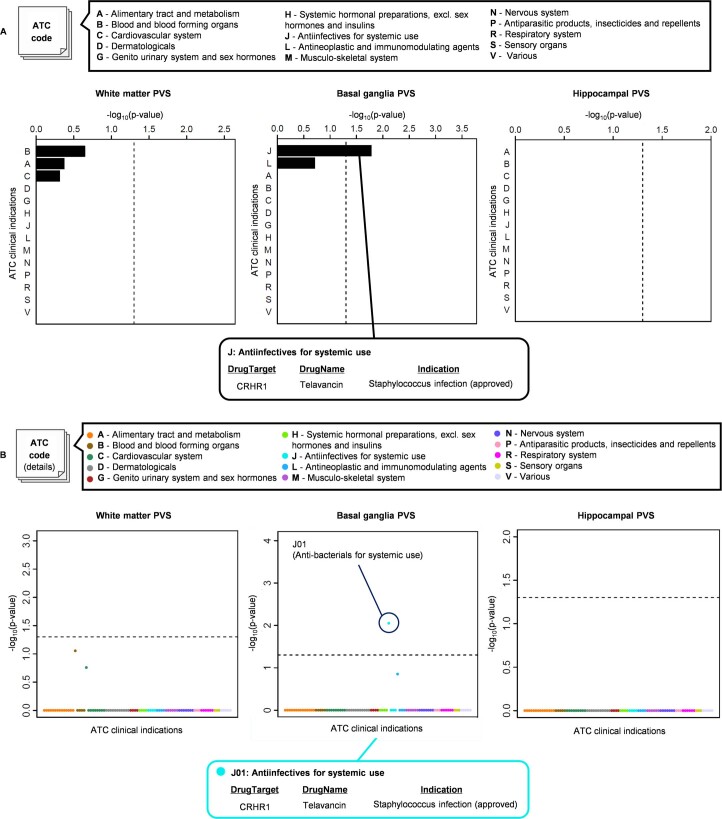
Enrichment of PVS genes in targets of drugs validated in other indications (Genome for REPositioning drugs, using ATC codes). We found a significant enrichment of BG-PVS genes in targets for antiinfectives for systemic use (ATC J01, OR = 252.4, P = 8.85×10^−3^), driven by *CRHR1* (chr17q21.31), a target for telavancin (**A, B**); of note, CRHR1 antagonists were shown to attenuate blood brain barrier permeability, cortical vascular hyperpermeability and tight junction disruption. ATC indicates Anatomical Therapeutic Chemical classification.

**Extended Data Fig. 8:**
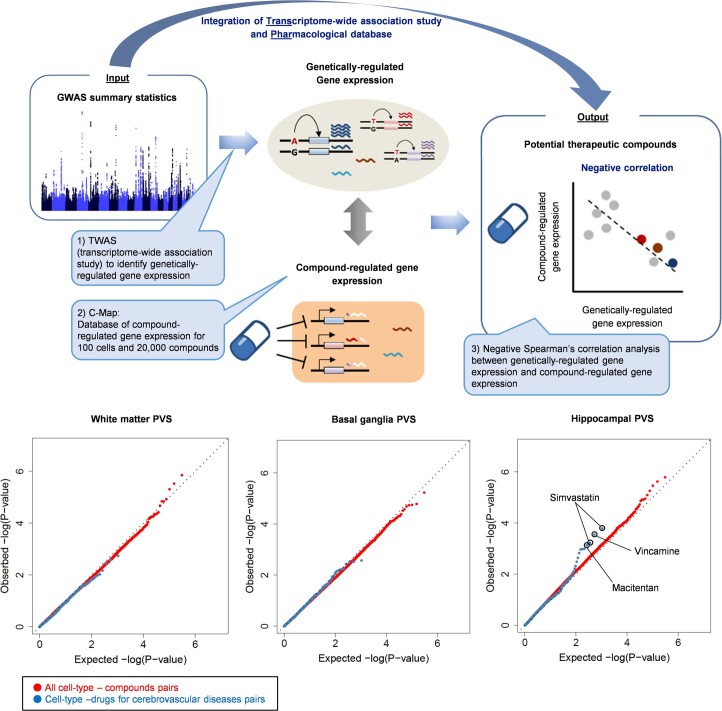
Enrichment of PVS genes in targets of drugs validated in other indications (Trans-Phar). We used the Integration of Transcriptome-wide Association Study and Pharmacological Database (Trans-Phar) software, leveraging TWAS on all GTEx v7 tissues and a database of compound-regulated gene expression (C-map) (Methods). A TWAS using FOCUS, which demonstrates fine-mapping of causal gene sets from TWAS results, and 27 tissues in the GTEx v7 database (corresponding to defined 13 tissue-cell-type categories assigned by the 27 tissues in GTEx v7 database and 77 LINCS CMap L1000 library cell types) was performed to identify up- and down-regulated genes in participants with extensive PVS burden, and select the top 10% genes with the highest expression variation. We observed significant enrichment of HIP-PVS in drugs for vascular diseases, including simvastatin (lipid-lowering drug, p = 1.64×10^−4^), vincamine (vasodilator increasing cerebral blood flow, p = 2.87×10^−4^), and macitentan (used for pulmonary arterial hypertension, p = 7.46×10^−4^). PVS indicates perivascular spaces.

Finally, we conducted an exploratory search for enrichment of PVS genes in targets of drugs validated in other indications (Methods). We found significant enrichment of BG-PVS genes in targets for anti-infectives, driven by *CRHR1* (chr17q21.31, target for telavancin), and for diseases of the nervous system, driven by *MAPT* (chr17q21.31, target for davunetide); and of HIP-PVS genes in targets for ear disease drugs, driven by *SERPIND1* (chr22q11.21, target for sulodexide, also used for venous thrombosis prevention; Extended Data Figs. 6 and 7). We also observed significant enrichment of TWAS-significant HIP-PVS genes in vascular disease drugs, including simvastatin, vincamine and macitentan (Extended Data Fig. 8).

## Discussion

In up to 40,095 participants from older population-based cohorts, we identified 24 genome-wide significant risk loci for extensive PVS burden, predominantly for WM-PVS, and six additional loci after accounting for other MRI markers of cSVD. Consistent with distinct risk factor profiles^2,10^, the genetic architecture of PVS differed across PVS locations, with WM-PVS showing the highest heritability and low genetic correlation with BG-PVS and HIP-PVS^1,2,16^. In line with the hypothesis that PVS is a marker of cSVD, moderate to high genetic correlation was observed with other MRI markers of cSVD, primarily for BG- and HIP-PVS. Pathway analyses highlight ECM structure and function, known to play an important role in cSVD^5,20,21^, and several loci include genes involved in the matrisome (ECM and associated proteins), perturbations of which were proposed as a convergent pathologic pathway in cSVD (*LAMC1*, *EFEMP1*, *COL4A2*, *SH3PXD2A*, *VWA2*)^5,21^. Several PVS risk loci (at *FOXF2*, *EFEMP1*, *KCNK2* and *NBEAL1-ICA1L*) are known risk loci for other cSVD features (WMH, SVS)^5,22,23^, and mutations in two MTAG genes cause monogenic SVD (at *COL4A1-COL4A2* and *STN1*)^24,25^.

PVS have been described early in life^7,26^, but their clinical significance at young ages is unknown. Our results suggest shared molecular mechanisms underlying PVS in young and older age. This corroborates recently described associations of WMH risk variants with changes in MRI-detected WM microstructure at age 20 yr (ref. ^5^). The significant enrichment of PVS risk loci in genes involved in early-onset leukodystrophies and expressed in fetal brain vascular endothelial cells supports involvement of developmental processes. In spontaneously hypertensive stroke-prone rats, closely modeling cSVD, intrinsic endothelial cell dysfunction was observed at birth, including reduced tight junctions, as well as altered oligodendrocyte maturation and myelination^27^. At the most significant WM-PVS locus in young adults, *OPA1* harbors mutations causing autosomal-dominant optical atrophy, sometimes associated with multiple sclerosis-like illness, parkinsonism and dementia^28^, and endothelial OPA1 plays an important role in developmental angiogenesis^29^. These observations corroborate epidemiological associations of early-life factors with cSVD severity in older age^30^.

The present effort has the largest East-Asian contribution compared with other large GWAS of MRI-defined phenotypes^31,32^, with over half of available WM-PVS loci reaching nominally significant, directionally consistent associations in the Japanese follow-up study. The prevalence of cSVD is higher in East-Asian than European populations^33^. Our results are an important initial step to establish the generalizability of cSVD genetic associations across ancestries. Efforts to further enhance the non-European contribution to MRI cSVD genomic studies, including in populations of African-ancestry in whom cSVD is also more frequent^34^, are of paramount importance.

The combination of PVS GWAS findings with TWAS and WES/WGS strongly supports putative causal genes. WM-PVS associates with lower *LPAR1* expression in vascular tissues. *LPAR1* (chr9q31.3), expressed in oligodendrocytes, encodes a receptor for lysophosphatidic acid, an extracellular signaling small lipid, and is involved in postnatal myelination and functional connectivity across brain regions^35^. An LPAR1 antagonist was found to attenuate brain damage after transient arterial occlusion, by decreasing inflammation^36^, and LPAR1 modulation may also impact neural regeneration^37^. Several drugs targeting LPAR1 are available (for example, the antidepressant mirtazapine^38^) or in development^39^. *WNT7A* (chr3p25.1) encodes a secreted signaling protein that targets the vascular endothelium, and was implicated in brain angiogenesis and blood brain barrier regulation^40^. Loss of Wnt7a/b function in mice results in severe WM damage^41^.

WM-PVS was associated with lower *ITGB5* (chr3q21.2) expression in whole blood. *ITGB5* encodes a beta subunit of integrin, and plays a central role in monogenic SVD^42^. Higher ITGB5 plasma levels were associated with decreased odds of cognitive impairment or dementia, lower brain amyloid burden and slower brain atrophy rates^43^. HIP-PVS was associated with lower expression of *LAMC1* (chr1q25.3, encoding Laminin gamma-1) in brain and higher expression in vascular tissues, while WES/WGS identified a splice donor variant at *LAMC1*. Laminins are ECM glycoproteins, and the major noncollagenous constituent of basement membranes. Genes encoding other basement membrane proteins (*NID2*, *COL4A1/2*) are implicated in cSVD^5,22^. Laminin regulates blood vessel diameter^44^ and blood brain barrier integrity and function^45^, and astrocytic laminin loss decreases expression of tight junction proteins and aquaporin-4 (AQP4)^45^, a key modulator of glymphatic flow in experimental models^7^.

Some genes point to complex pleiotropic mechanisms. At chr2q33.2, also associated with WMH, SVS, Alzheimer’s disease and caudate volume^5,23,46,47^, BG-PVS was associated with higher expression of *ICA1L* in brain tissues and of *NBEAL1* in vascular tissues, similar to TWAS of WMH and SVS^5,22^. *ICA1L* (encoding islet cell autoantigen-1-like and predominantly expressed in endothelial cells) harbors mutations causing juvenile amyotrophic lateral sclerosis^48^, while *NBEAL1* (encoding neurobeachin-like 1 protein) modulates low-density lipoprotein (LDL)-receptor expression^49^.

Our study points to an important involvement of solute carriers (SLCs), the largest family of transporters and candidates for drug target development^50^, in PVS pathophysiology. The most significant PVS risk variants involve an intronic haplotype of *SCL13A3*, encoding a plasma membrane Na^+^/dicarboxylate cotransporter expressed in kidney, astrocytes and choroid plexus^51^. Mutations in *SLC13A3* cause acute reversible leukoencephalopathy with increased urinary alpha-ketoglutarate^51^, where *SLC13A3* loss-of-function may affect elimination of organic anions and xenobiotics from the cerebrospinal fluid (CSF)^51^. At the same locus (Supplementary Fig. 1), other genome-wide significant variants are located near *SLC2A10*, harboring mutations causing arterial tortuosity syndrome^52^, described to be associated with PVS burden and cSVD^53^. WM-PVS was associated with lower *SLC20A2* expression in brain tissue. *SLC20A2*, involved in phosphate transport, harbors loss-of-function mutations causing idiopathic familial BG calcification, a neurodegenerative disorder with inorganic phosphate accumulation in the ECM^54^. Given their role in CSF secretion and substance transport at the blood–CSF barrier^55^, SLCs could be involved in interstitial fluid accumulation adjacent to the PVS^56^.

Consistent with other SVD phenotypes, we observed evidence for a causal association of blood pressure with PVS. Experimental work suggests that the perivascular pump becomes less efficient with increasing blood pressure, reducing net forward flow in the PVS. These effects were found to be larger at more distal locations, where arteries have thinner and less muscular walls^57^. Such hemodynamic and anatomic differences^1,2,18^ could, perhaps, at least partly explain the more significant association of blood pressure with BG-PVS and HIP-PVS compared with WM-PVS. In contrast, WM-PVS were previously found to be associated with CAA^11^ and with higher brain amyloid deposition on positron emission tomography, across the clinical spectrum of CAA^12^. The updated Boston Criteria (v.2.0) for CAA include severe WM-PVS as a new diagnostic criterion^13^.

The clinical relevance of PVS is strongly supported by the significant genetic correlation of BG-PVS and HIP-PVS with any stroke and ischemic stroke and robust evidence for a possible causal association of BG-PVS and HIP-PVS with any stroke, ischemic stroke and SVS, accounting for blood pressure. The nominally significant genetic correlation of BG-PVS and HIP-PVS with (deep) ICH, based on smaller GWAS and thus less statistical power, is also consistent with epidemiological findings^10^. Considering the association of HIP-PVS with lower *LAMC1* expression in brain, it is striking to note that conditional knock-out of laminin in astrocytes leads to deep ICH in mice^58^. This is reminiscent of known associations of variants in *COL4A1/A2*, encoding another basement membrane protein, with monogenic and multifactorial deep ICH^46,59^.

Significant enrichment of PVS genes in targets of drugs validated or under investigation for vascular and cognitive disorders (for example, telavancin and davunetide) highlights the potential of PVS genetics for cSVD drug discovery.

To our knowledge, this is the first study exploring the genetic determinants of PVS, using a comprehensive gene-mapping strategy and extensive bioinformatics follow-up. We acknowledge limitations. To account for heterogeneity in PVS quantification methods, we pragmatically dichotomized PVS variables based on the top quartile of the distribution, which may be less powerful than continuous measures. This may have been most prominent for BG-PVS, for which the genetic correlation pattern between CHARGE and UKB was low, in contrast with WM-PVS and HIP-PVS. Reassuringly, loci identified using dichotomous PVS phenotypes were also associated with continuous PVS burden in studies where computational methods were available (UKB, i-Share, Nagahama), mostly with more significant *P* values. A conservative approach will also have helped minimize the effect of accidentally including WMH in the PVS measures, a problem which some computational PVS methods have not yet overcome. Strikingly, 67% of WM-PVS loci were associated at least nominally with WM-PVS in one or both follow-up cohorts, despite considerably smaller samples and distinct age and ancestry, with consistent directionality. This suggests that our genomic discovery approach, although likely conservative, led to robust findings. With increasing development of artificial intelligence-based computational methods for PVS quantification, future genomic studies will likely have even greater power to detect genetic associations, to enable studying the genomics of total PVS volume, accounting for differences in individual PVS volume, width, length, shape^60^, density, location and anatomical predominance, and to run sex-specific analyses.

In conclusion, in this gene-mapping study of PVS, one of the earliest MRI markers of cSVD, we describe 24 genome-wide significant risk loci, with six additional loci in secondary multivariate analyses accounting for other cSVD markers. Our findings provide insight into the biology of PVS across the adult lifespan and its contribution to cSVD pathophysiology, with potential for genetically informed prioritization of drug targets for prevention trials of cSVD, a major cause of stroke and dementia worldwide.

## Methods

### Study design

This study complies with all relevant ethical regulations, and all participants gave written, informed consent. Analyses were performed on stroke-free participants from 22 population-based cohorts (18 for the GWAS meta-analysis), taking part in UKB, the CHARGE consortium and the BRIDGET initiative. Institutional review boards approved individual studies: UKB (National Research Ethics Service Committee North West–Haydock), 3C-Dijon (Ethical Committee of the University Hospital of Kremlin-Bicêtre), Austrian Stroke Prevention Study and Austrian Stroke Prevention Family Study (ASPS/ASPS-Fam) (Ethics Committee of the Medical University of Graz), Epidemiology of Dementia in Singapore Study (EDIS) (the Singapore Chinese Eye Study/Singapore Malay Eye Study-2, Singapore Eye Research Institute and the National Healthcare Group Domain-Specific Review Board), Framingham Heart Study (FHS) (Institutional Review Board of Boston University Medical Center), Investigating Silent Strokes in Hypertensives Study (ISSYS) (Comité de ética de investigacion con medicamentos, Hospital Universitari Vall d’Hebron), Lothian Birth Cohort 1936 (LBC1936) (Lothian and Scottish Multicentre Research Ethics Committees), Northern Manhattan Study (NOMAS) (Columbia University Medical Center Institutional Review Board and the University of Miami Institutional Review Board), Rotterdam Study I, II and III (RS-I, RS-II and RS-III) (Ministry of Health, Welfare, and Sport of the Netherlands), Study of Health in Pomerania (SHIP) (SHIP-2, SHIP-Trend Batch 1 and 2, Ethics Commission of the University of Greifswald), i-Share study (Comités de Protection des Personnes (CPP) Sud-Ouest Outre-Mer III, Sydney Memory and Ageing Study (MAS) (Ethics Committees of the University of New South Wales, South-Eastern Sydney, and the Illawarra Area Health Service), Older Australian Twins Study (OATS) (Ethical Committees of the Australian Twin Registry, the University of New South Wales, the University of Melbourne, the Queensland Institute of Medical Research, and the South-Eastern Sydney and Illawarra Area Health Service) and the Nagahama Study (Ethics Committee of Kyoto University Graduate School of Medicine and the Nagahama Municipal Review Board) (Supplementary Table 1). Characteristics of study participants are provided in Supplementary Tables 1–3 and 26 and Supplementary Fig. 5.

### PVS burden definition

PVS were defined as fluid-filled spaces with a signal identical to that of CSF, of round, ovoid or linear shape depending on the slice direction, with usually a maximum diameter smaller than 3 mm, no hyperintense rim on T2-weighted or FLAIR sequences, and located in areas supplied by perforating arteries^3^. In most CHARGE cohorts, visual semiquantitative rating scales were used to quantify PVS burden. As different scales were used across studies, we dichotomized PVS burden into ‘extensive PVS burden’ versus the rest, defined by a cut-off closest to the top quartile of the semiquantitative scale distribution within each cohort (Supplementary Tables 2, 27 and 28). This cohort-specific threshold definition was chosen because (1) small PVS counts are very sensitive to MRI field strength and less prominently associated with age and vascular risk factors^61^; (2) extreme burden of other MRI markers of cSVD (for example, extensive WMH burden within the top quartile of the distribution) was previously shown to facilitate the identification of genetic variants underlying cSVD^62^; and (3) PVS burden is highly dependent on participant characteristics, especially age, PVS quantification methods and image acquisition parameters. In RS-III and in UKB, a recently developed automated method was used to quantify the number of PVS (Supplementary Table 27), dichotomized according to the same cut-off (top quartile). For sensitivity analyses, we also compared results obtained in UKB with the dichotomized and continuous (log-transformed) PVS variables.

### Covariates and descriptive variables

Intracranial volume (sum of gray matter, WM and CSF volumes) was available in all studies except ASPS, where brain parenchymal fraction was used (ratio of brain parenchymal tissue volume to total volume within the surface contour of the whole brain). Other covariates are described in Supplementary Table 1.

### Genotyping and imputation

Genome-wide genotypes were imputed to the 1000G project (1000G pIv3) or the Haplotype Reference Consortium reference panels (Supplementary Table 3).

### PVS genome-wide association analyses in individual cohorts

Ancestry-specific logistic regression analyses with an additive genetic model were performed, adjusting for age, sex (genetically determined) and intracranial volume (or brain parenchymal fraction for ASPS), principal components of population stratification, and study site.

As sensitivity analyses, we ran linear mixed models in UKB, (1) using the log-transformed (log(variable + 1)) continuous PVS measurements, adjusting for the same covariates as above; (2) generating residuals adjusting for the same covariates and then dichotomizing the residuals (instead of adjusting for covariates after dichotomization).

### PVS genome-wide association meta-analyses

We performed quality control in each study following the recommendations of Winkler et al.^63^. Analyses were done on autosomal biallelic markers. Duplicate markers were removed, marker names and alleles were harmonized across studies, and PZ-plots, quantile–quantile plots and allele frequency plots were constructed^63^. In each study, rare variants (MAF < 0.01) and variants with low imputation accuracy (*R*², oevar_imp or info score < 0.5) or extensive effect size values (β > 5 or β < −5) were removed. We reported the number of SNPs passing quality control for each study (Supplementary Table 4). GWAS were run within each cohort using logistic regression (or linear regression for sensitivity analyses), using software described in Supplementary Table 3. We then conducted GWAS meta-analyses across participating cohorts in METAL, using sample size-weighted meta-analysis as PVS were measured on different scales. Meta-analyses were conducted within each ancestry (European (EUR), Asian (ASN), African-American (AA), Hispanic (HISP)) using METAL (https://github.com/statgen/METAL), followed by meta-analyses across ancestries. Ancestry was genetically inferred using principal components of population stratification (Supplementary Tables 1 and 3). Genomic control was applied to each study-specific GWAS with a genomic inflation factor greater than 1.00. Variants with an effective allele count (twice the product of MAF, imputation accuracy and number of participants with extensive PVS) < 10 and significant heterogeneity (*P*_Het_ < 5.0 × 10^−8^) were excluded from the meta-analysis. We performed LD-clumping, sorting the genome-wide significant SNPs by *P* value, keeping the most significant SNP and removing SNPs with an *r*² > 0.1 within 1 megabase (Mb). Only variants present in at least half of participants of the final meta-analysis were used to construct quantile–quantile and Manhattan plots. In secondary analyses, we ran inverse variance-weighted meta-analyses to obtain effect estimates and standard errors for follow-up bioinformatics analyses.

### Conditional and joint multiple-SNP analysis

We used GCTA-COJO^64^ to perform conditional and joint multiple-SNP analysis of PVS GWAS summary statistics, to identify secondary association signals at each of the genome-wide significant loci within 1 Mb of the lead SNP. We used European GWAS summary statistics as recommended to avoid population stratification. This method relied on a stepwise selection procedure to select SNPs based on the conditional *P* values, and the joint effects of all selected SNPs after optimization of the model were estimated^64^. We used genotypes of 6,489 unrelated participants of European ancestry from the 1000G-imputed 3C-Dijon study data for LD correction. We performed haplotype association analyses on the six independent lead variants at chr20q13.12 (Supplementary Table 5b).

### Cross-ancestry meta-regression of GWAS

We conducted cross-ancestry meta-analyses using MR-MEGA^65^, which uses meta-regression to model allelic effects, including axes of genetic variation as covariates in the model.

### Gene-based analyses

We performed gene-based analyses on European PVS GWAS meta-analyses. We included variants within 10 kilobase (kb) of the 3′ and 5′ untranslated regions (UTRs) of a gene to capture regulatory variants. We used the MAGMA software implemented in FUMA^66^ to perform a gene-based association study, including 19,037 protein-coding genes. This method is based on a multiple linear principal components regression model. Gene-wide significance was defined at *P* < 2.63 × 10^−6^. We also performed gene-based tests using VEGAS2 (ref. ^67^), including 18,371 autosomal genes, leading to a gene-wide significance at *P* < 2.72 × 10^−6^. Genes were considered in the same locus if they were within 200 kb of each other.

### PVS heritability estimates

We used LD-score regression (ldsc package https://github.com/bulik/ldsc/) to estimate the heritability of extensive PVS burden in each location, overall and, in secondary analyses, separately, in CHARGE and UKB.

### Multi-trait GWAS with PVS and other MRI markers of cSVD

We conducted a joint analysis of summary statistics from GWAS of PVS, WMH and lacunes using MTAG^68^, with the expectation to gain in power because of the genetic correlation between these MRI markers of cSVD. MTAG is a generalization of inverse variance-weighted GWAS meta-analysis of two or more traits, which accounts for sample overlap between GWAS results for different traits by employing LD-score regression. MTAG is based on the assumption that all SNPs share the same variance–covariance matrix of effect sizes across traits. We prioritized variants with a *P* < 5 × 10^−8^ in the PVS MTAG analysis and *P* < 0.05 in the univariate PVS GWAS, which showed greater significance for association with PVS in MTAG than in univariate analyses for PVS, WMH and lacunes.

### PVS next-generation sequencing association analyses

Using WES data and exome content of WGS data in 19,010 participants from UKB and BRIDGET, of whom 4,531, 4,424 and 4,497 had extensive PVS in WM, BG and HIP, respectively, we performed a whole-exome association study to identify (rare) exonic variants associated with extensive PVS (Supplementary Tables 1 and 29).

### Follow-up of findings across lifespan and ancestries

We explored associations of WM-PVS and BG-PVS risk variants identified in the GWAS meta-analysis with these phenotypes in young adults (i-Share study, *N* = 1,748, mean age 22.1 ± 2.3 yr) and in older Japanese population-based cohort participants (Nagahama study, *N* = 2,862, 68.3 ± 5.3 yr; Supplementary Tables 1 and 3). In each study, we used both quantitative PVS measurements derived from a computational artificial intelligence-based method (Supplementary Tables 27 and 28) and dichotomized PVS burden (top quartile of PVS distribution; Supplementary Table 2). HIP-PVS data were not available. Continuous PVS measurements were log-transformed (log(variable + 1)) to obtain a normal distribution.

In i-Share participants of European ancestry, we also explored the association of WM-PVS with a wGRS of WM-PVS burden derived from the 21 independent genome-wide significant SNPs identified in the European GWAS meta-analysis (*r*² < 0.10 based on the 1000G European reference panel). SNPs were weighted by the SNP effect sizes in the European GWAS meta-analysis (for the allele associated with larger PVS burden); the wGRS was rescaled (rwGRS) so that one unit of the wGRS corresponds to one additional WM-PVS risk allele. We tested for significant modifying effects of age on associations with WM-PVS for the three genome-wide significant WM-PVS loci in young adults (at chr2p16.1, chr3q29 and chr20q13.12). We collected effect estimates and standard errors for the lead SNPs at these three loci in each individual cohort, and fitted a meta-regression of the lead SNPs’ effect sizes onto an intercept and age. Meta-regression analysis was performed using Metafor^69^, and any statistical evidence of linear association was corrected for multiple testing (*P* < 0.05/3 = 1.7 × 10^−2^).

In Nagahama we explored the association of WM-PVS with a rwGRS of WM-PVS burden, including the 14 available independent SNPs identified in the European GWAS meta-analysis (*r*² < 0.10 based on 1000G Japanese reference panel); SNPs were weighted by the SNP effect sizes in the European GWAS meta-analysis.

### Shared genetic variation with other phenotypes

In the European ancestry meta-analysis, we explored shared genetic variation with vascular and neurological phenotypes: (1) putative risk factors (SBP, DBP, pulse pressure, body mass index, high-density lipoprotein cholesterol, LDL cholesterol, triglycerides, type 2 diabetes and sleep patterns); (2) other MRI markers of brain aging (WMH burden, covert MRI-defined brain infarcts and lacunes, and hippocampal, nucleus accumbens, amygdala, caudate nucleus, pallidum and putamen volumes); and (3) the most common neurological conditions previously reported to be associated with PVS, namely stroke (any stroke, any ischemic stroke, large artery stroke, cardio-embolic stroke, SVS, ICH), Alzheimer’s disease and Parkinson’s disease (Supplementary Table 30).

We explored whether genome-wide significant PVS risk loci (lead variants or in LD with *r*² > 0.9, based on the 1000G European reference panel) were associated with these traits. A *P* value threshold <3.3 × 10^−5^, correcting for 21 independent phenotypes, three PVS locations and 24 independent loci tested, was used (Supplementary Table 30). We performed a colocalization analysis using COLOC to search for evidence for a single causal variant between PVS and the other phenotypes, a posterior probability (PP4) > 75% supporting a single causal variant for both traits^70^.

Second, we used LD-score regression (ldsc package: https://github.com/bulik/ldsc/) to estimate the genetic correlation of extensive PVS burden with these phenotypes (*P* < 7.9 × 10^−4^ was used as a significance threshold, correcting for 21 phenotypes and three PVS locations). To decrease potential bias due to poor imputation quality, the summary statistics were filtered to the subset of HapMap3 SNPs for each trait. In secondary analyses, we estimated genetic correlation of PVS burden with the same traits separately in CHARGE and UKB.

We used FUMA to obtain extensive functional annotation for genome-wide significant SNPs and to identify SNPs associated with other traits at genome-wide significance from the GWAS catalog^66^.

### MR

We used an MR approach to explore the possible causal relation of putative risk factors (vascular risk factors and sleep patterns) with extensive PVS burden, and of extensive PVS burden with neurological traits (stroke, Alzheimer’s disease and Parkinson’s disease).

We used the GSMR method implemented in GCTA^71^. Summary statistics were clumped using 1000G-imputed 3C-Dijon study data (*r*² < 0.05 and *P* < 5 × 10^−8^) using only SNPs with MAF > 0.01. The heterogeneity in independent instrument (HEIDI)-outlier method was used to remove genetic instruments that showed pleiotropic effects on both the exposure and the outcome.

For (at least) nominally significant GSMR associations, we conducted secondary MR analyses using both TwoSampleMR and RadialMR^72,73^. Only independent SNPs (*r*^2^ < 0.01 based on 1000G European, window size = 1 Mb) reaching *P* < 5 × 10^−8^ in the primary meta-analysis were included as recommended. Effect estimates (β values) and SE values were derived from the inverse variance-weighted GWAS meta-analyses. With TwoSampleMR, we estimated the effect of each exposure on each outcome using weighted median, random-effect inverse variance weighting (IVW) and MR-Egger. In addition, we confirmed the directionality of the observed associations with the Steiger test^74^. With RadialMR (https://github.com/WSpiller/RadialMR), the putative causal effect of each exposure on each outcome was estimated using the fixed-effect IVW method using the modified second-order inverse variance weight^73^. Cochran’s *Q* statistic was used to test for the heterogeneity (*P* < 0.05) due to horizontal pleiotropy^73^. We excluded outlier SNPs, identified by regressing the predicted causal estimate against the inverse variance weights^73^, and re-ran IVW tests, as well as MR-Egger regression, assessing heterogeneity with Rücker’s *Q*′ statistic^73^. When the ratio of *Q*′ (Egger) on *Q* (IVW) (*Q*_R_) was close to 1, indicating that both IVW and MR-Egger models fit the data equally, we selected the IVW model. We formally ruled out horizontal pleiotropy when the MR-Egger intercept after exclusion of outliers was nonsignificant (*P* ≥ 0.05). To account for potential residual correlated pleiotropy, we used MR-CAUSE^75^. Finally, we explored the association between genetic liability to PVS and stroke, conditioning on blood pressure (SBP and DBP separately), by running multivariable MR analyses using TwoSampleMR^72^. A *P* < 1.19 × 10^−3^, correcting for 14 independent phenotypes and the three PVS locations, was considered significant.

### Pathway analyses

We used MAGMA gene set analyses (in FUMA^66^) to identify pathways overrepresented in the associations. We identified genes associated with extensive PVS burden and estimated the correlation between genes. The *P* values and gene correlation matrix were used in a generalized least squares model. A *P* < 3.2 × 10^−6^ correction for 15,496 gene sets was considered significant. As a sensitivity analysis, we used VEGAS2Pathway^76^, which aggregates association strengths of individual markers into prespecified biological pathways using VEGAS-derived gene association *P* values for extensive PVS burden, with an empirical significance threshold of *P* < 1 × 10^−5^ (accounting for 6,213 correlated pathways).

### Enrichment analyses in OMIM and COSMIC genes

Using hypergeometric tests, we performed enrichment analyses of genes within 1 Mb, 100 kb or 10 kb of the lead variants, but also of genes within 10 kb of the lead variants with intragenic variants, and genes within 10 kb of the genetic loci with intragenic lead variants. We used the rest of the protein-coding genome as a reference. We performed the analysis first combining loci of all PVS locations, and second including only WM-PVS loci. We searched for an enrichment in different gene groups from the OMIM database^77^, including PVS (‘perivascular space’ OR ‘virchow-robin space’), WMH (‘leukoaraiosis’ OR ‘white matter lesion*’* OR ‘white matter hyperintensities’) and leukodystrophy (‘leukodystrophy*’* OR ‘leukoencephalopathy’) genes. We also searched for an enrichment of genes involved in glioma and glioblastoma, identified in the Catalog Of Somatic Mutations In Cancer (COSMIC) (https://cancer.sanger.ac.uk).

### TWAS

We performed TWAS using TWAS-Fusion^78^, to identify genes whose expression is significantly associated with PVS burden without directly measuring expression levels. We restricted the analysis to tissues considered relevant for cerebrovascular disease, and used precomputed functional weights from 22 publicly available gene expression reference panels from blood, arterial, brain and peripheral nerve tissues (Fig. 4). TWAS-Fusion was then used to estimate the TWAS association statistics between predicted gene expression and PVS burden by integrating information from expression reference panels (SNP expression weights), GWAS summary statistics (SNP PVS effect estimates) and LD reference panels (SNP correlation matrix). Transcriptome-wide significant genes (eGenes) and the corresponding expression quantitative trait loci (eQTLs) were determined using Bonferroni correction (*P* < 3.93 × 10^−6^, correcting for 4,235 genes tested and three PVS locations). eGenes were then tested in conditional analyses as implemented in TWAS-Fusion. Next, we performed a genetic colocalization analysis of gene expression and PVS burden for each conditionally significant gene (*P* < 0.05) using COLOC^70^, to estimate the posterior probability of a shared causal variant between the gene expression and the trait (PP4 ≥ 0.75). Gene regions with eQTLs not reaching genome-wide significance in association with PVS, and not in LD (*r*^2^ < 0.01) with the lead SNP for genome-wide significant PVS risk loci, were considered as novel.

### Cell type enrichment analysis

We conducted a cell type enrichment analysis using Single cell Type Enrichment Analysis for Phenotypes (https://github.com/erwinerdem/STEAP/). This is an extension to CELLECT and uses S-LDSC, MAGMA and H-MAGMA for enrichment analysis. PVS GWAS summary statistics were munged. Then, expression specificity profiles were calculated using human and mouse single-cell RNA sequencing databases (PsychENCODE DER-22, GSE67835, GSE101601, DroNc Human Hippocampus, Allen Brain Atlas MTG and LNG, Mousebrain, Tabula Muris, Descartes Human Cerebrum and Cerebellum; Supplementary Table 24). Cell type enrichment was calculated with MAGMA, H-MAGMA (incorporating chromatin interaction profiles from human brain tissues in MAGMA) and stratified LD-score regression. *P* values were corrected for the number of independent cell types in each database.

### Lifetime brain gene expression profile

We studied the lifetime expression of genes identified in the TWAS-COLOC analysis, and the three genes associated with WM-PVS burden in both the old and young populations, to search for developmental processes. We used a public database (https://hbatlas.org/) comprising genome-wide exon-level transcriptome data from 1,340 tissue samples from 16 brain regions (cerebellar cortex, mediodorsal nucleus of the thalamus, striatum, amygdala, hippocampus and 11 neocortex areas) of 57 postmortem human brains, from embryonic development to older adults of different ancestries.

### Enrichment in drug target genes

We used the GREP (Genome for Repositioning)^79^ software tool, which quantifies an enrichment of gene sets from GWAS summary statistics in drugs of certain Anatomical Therapeutic Chemical Classification (ATC) classes, or indicated for some ICD10 (10th revision of the International Statistical Classification of Diseases and Related Health Problems) disease categories, and captures potentially repositionable drugs targeting the gene set. Genes with false discovery rate FDR q < 0.1 in MAGMA were used for enrichment analyses (in GREP) of target genes for approved or investigated drugs curated in DrugBank and the Therapeutic Target Database.

We used the Trans-Phar (integration of TWAS and Pharmacological database) software to identify drug target candidates in a specific tissue or cell type^80^, using first FOCUS to identify up- and downregulated genes in participants with extensive PVS burden, followed by a negative Spearman’s rank correlation analysis between the gene expression (*Z*-score) of the top 10% genes with the highest expression variation and the LINCS CMap L1000 library database (Extended Data Fig. 8).

### Reporting summary

Further information on research design is available in the Nature Portfolio Reporting Summary linked to this article.

## Online content

Any methods, additional references, Nature Portfolio reporting summaries, source data, extended data, supplementary information, acknowledgements, peer review information; details of author contributions and competing interests; and statements of data and code availability are available at 10.1038/s41591-023-02268-w.

## Supplementary information

Supplementary Information Supplementary figures.

Reporting Summary

Supplementary Tables Supplementary tables.

## Data Availability

Genome-wide summary statistics for the European and cross-ancestry meta-analysis generated and analyzed during the current study are deposited on the GWAS Catalog (study code GCST90244151-GCST90244156). As for other meta-analyses of GWAS or sequencing data, individual cohort data are subject to controlled access, for privacy and legal issues (national and European regulations, including GDPR). This applies to all participating cohorts (cohorts included in the meta-analyses and follow-up cohorts). UKB data (GWAS and sequencing) are accessible by submitting an application to the UKB portal (this research has been conducted under Application Number 23509). We used publicly available data for analyses described in this manuscript, including data from GTEx (https://gtexportal.org/home/), the Gusev laboratory (http://gusevlab.org/projects/fusion/), the CommonMind Consortium (https://www.nimhgenetics.org/resources/commonmind), the Netherlands Twin Registry (https://tweelingenregister.vu.nl/), the Young Finns Study (https://youngfinnsstudy.utu.fi/), OMIM (https://www.omim.org/), OMIM genes description are publicly available: GFAP (https://www.omim.org/entry/137780); SLC13A3 (https://www.omim.org/entry/618384); PNPT1 (https://www.omim.org/entry/610316), COSMIC (https://cancer.sanger.ac.uk), RNA sequencing datasets: PsychENCODE DER-22 (www.ncbi.nlm.nih.gov/geo/, accession code GSE97942), GSE67835 (www.ncbi.nlm.nih.gov/geo/, accession code GSE67835), GSE101601, DroNc_Human Hippocampus (https://www.gtexportal.org/home/datasets), Allen Brain Atlas (http://portal.brain-map.org/), Descartes_Human (https://descartes.brotmanbaty.org/), Mousebrain (http://mousebrain.org/), Tabula Muris (https://tabula-muris.ds.czbiohub.org/). All other data supporting the findings of this study are available within the article, the supplementary information or the supplementary data files.

## Code availability

The PVS quantification method used in the Nagahama Study is available using this link: https://github.com/pboutinaud/SHIVA_PVS. We used publicly available data from METAL (https://github.com/statgen/METAL), GCTA-cojo (https://yanglab.westlake.edu.cn/software/gcta/#COJO), FUMA (https://fuma.ctglab.nl/), MAGMA (https://ctg.cncr.nl/software/magma), LD-Score Regression (https://github.com/bulik/ldsc/), MTAG (https://github.com/JonJala/mtag), coloc (https://chr1swallace.github.io/coloc/) R package, RadialMR (https://github.com/WSpiller/RadialMR), TwoSampleMR (https://mrcieu.github.io/TwoSampleMR/), STEAP (https://github.com/erwinerdem/STEAP/), CELLECT (https://github.com/perslab/CELLECT), S-LSC (https://github.com/bulik/ldsc), H-MAGMA (https://github.com/thewonlab/H-MAGMA), HBT (https://hbatlas.org/). Drug discovery analysis was conducted using the following publicly available tools: GREP (https://github.com/saorisakaue/GREP), Trans-Phar (https://github.com/konumat/Trans-Phar).
