# Supplementary material for: Genomics of perivascular space burden unravels early mechanisms of cerebral small vessel disease
Source: Nat Med. 2023 Apr 17;29(4):950–62. doi: 10.1038/s41591-023-02268-w (PMC10115645; doi:10.1038/s41591-023-02268-w)
Supplement: Supplementary file 1 — Supplementary figures. [file 41591_2023_2268_MOESM1_ESM.pdf]

nature medicine

Article https://doi.org/10.1038/s41591-023-02268-w

Genomics of perivascular space burden unravels early mechanisms of cerebral small vessel disease

In the format provided by the authors and unedited

Genomics of perivascular space burden unravels early mechanisms of cerebral small vessel disease

SUPPLEMENTARY DATA

Table of contents

SUPPLEMENTARY FIGURES **2**
1) Supplementary Fig. 1 : Regional association plots 2
2) Supplementary Fig. 2 : Association of WM-PVS SNPs with WM-PVS burden in the i-Share cohort (N=1,748) 8
3) Supplementary Fig. 3 : Meta-regression with age of the three genome-wide significant WM-PVS loci that also showed significant associations with WM-PVS in young adults 9
4) Supplementary Figure 4. Brain expression pattern across the lifespan of genes near genome-wide significant PVS loci that are also identified in the TWAS or that are the nearest genes of variants showing an association with PVS already in young adults 11
4.1. Genes in WM-PVS loci 11
4.2. Genes in BG-PVS loci 13
4.3. Genes in HIP-PVS loci 14
5) Supplementary Figure 5. PVS distribution 15

SUPPLEMENTARY FIGURES

1) Supplementary Fig. 1 : Regional association plots

Each circle indicates a single-nucleotide polymorphism (SNPs) with a color scale corresponding to the r^2 value for that SNP and the top SNP from 1000 Genomes. Purple diamonds indicate the SNPs with the strongest association. Estimated recombination from 1000 Genomes are indicated with blue lines. The bottom panels show the relative position of genes within each locus.

1.1. Perivascular spaces in white matter

Chr20q13.12

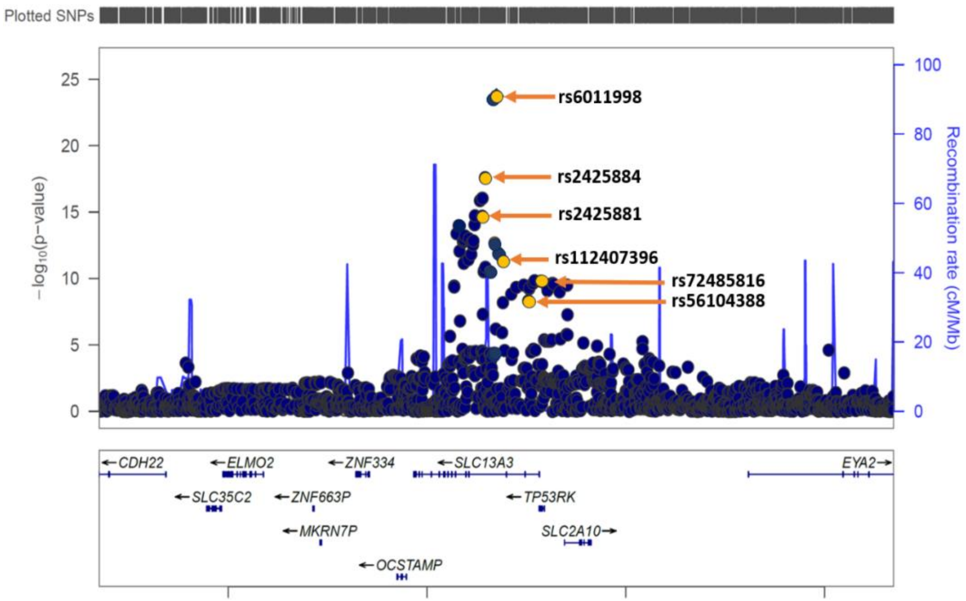

Regional plot of the chr20q13.13 locus with 6 independent signals identified by GCTA-COJO (orange).

Chr3p25.1

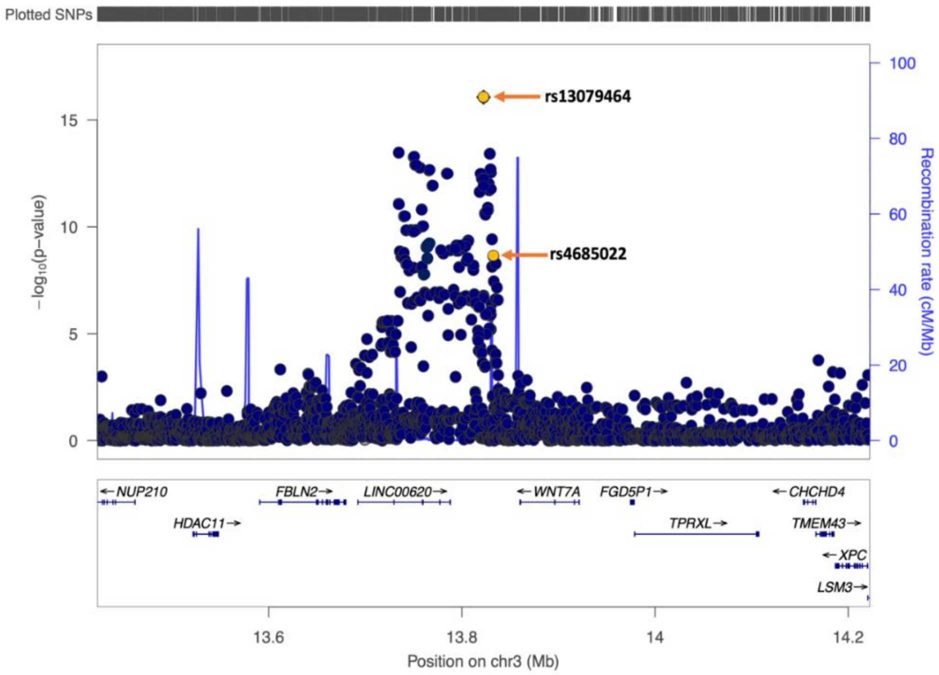

Chr 9q31.3

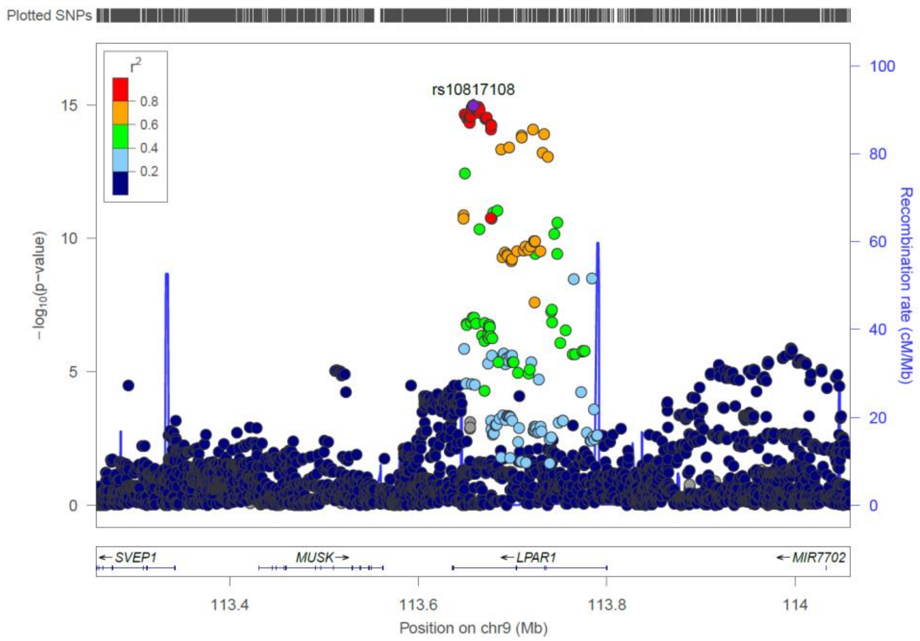

Chr 3q21.2

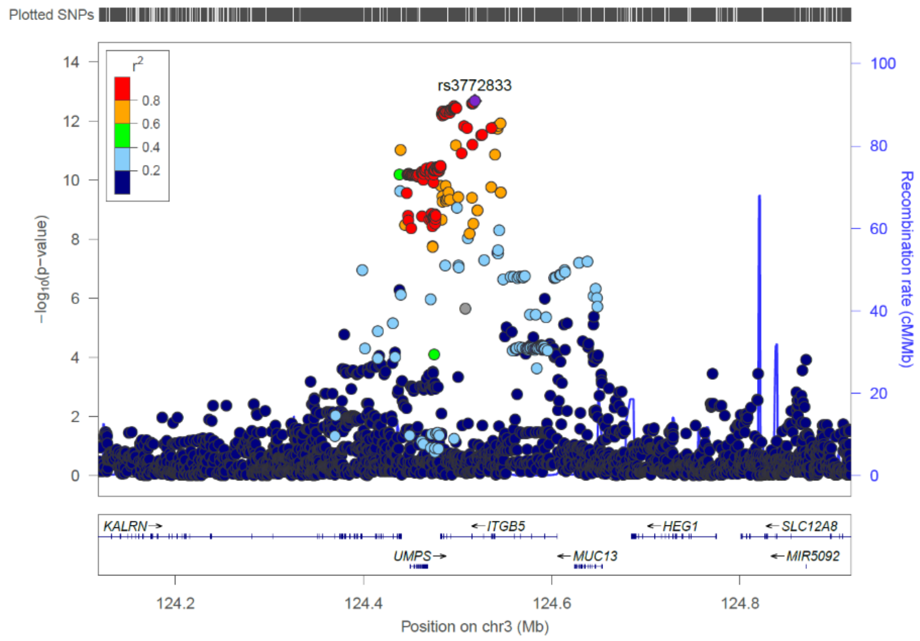

Chr 1q41

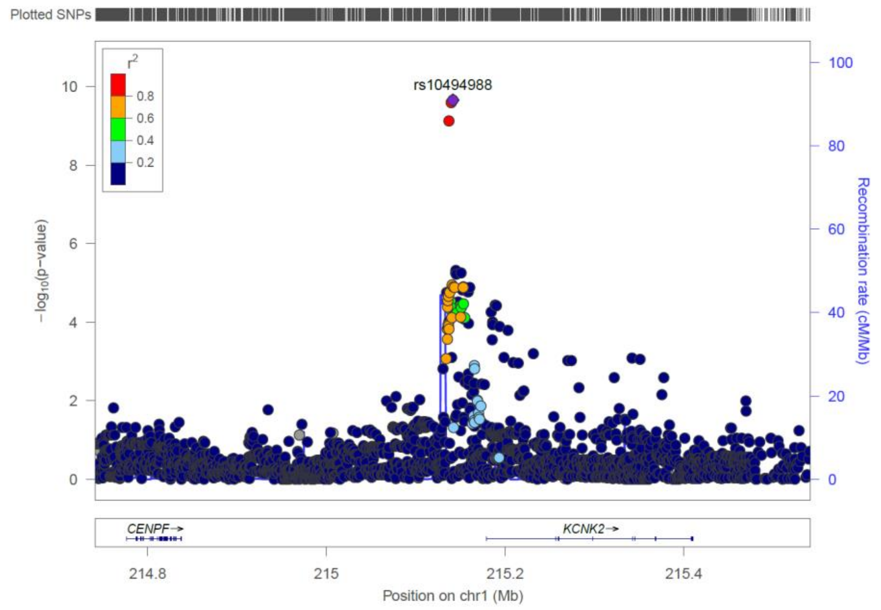

Chr 15q25.3

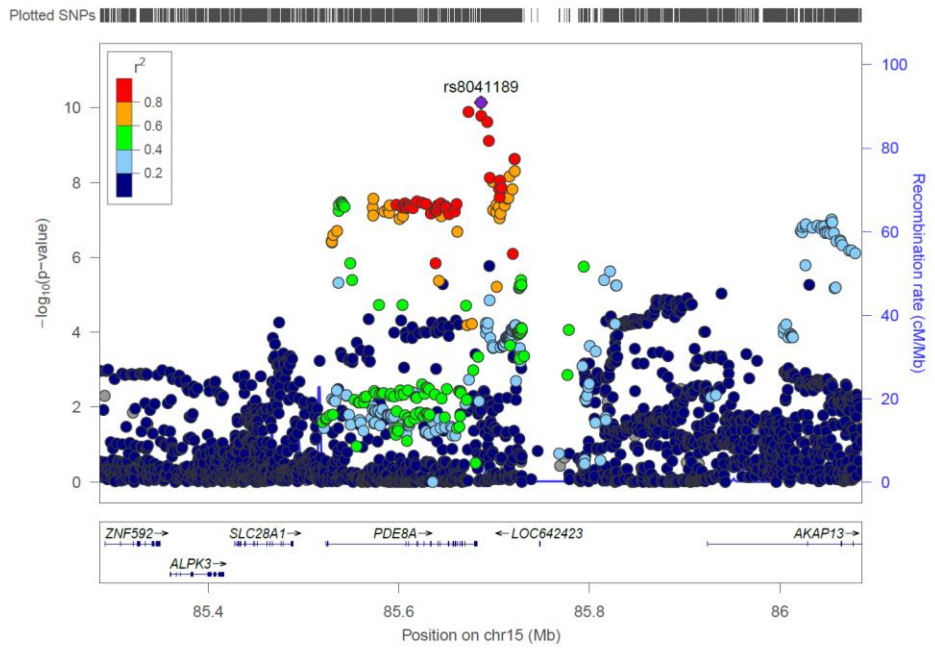

Chr 2p16.1

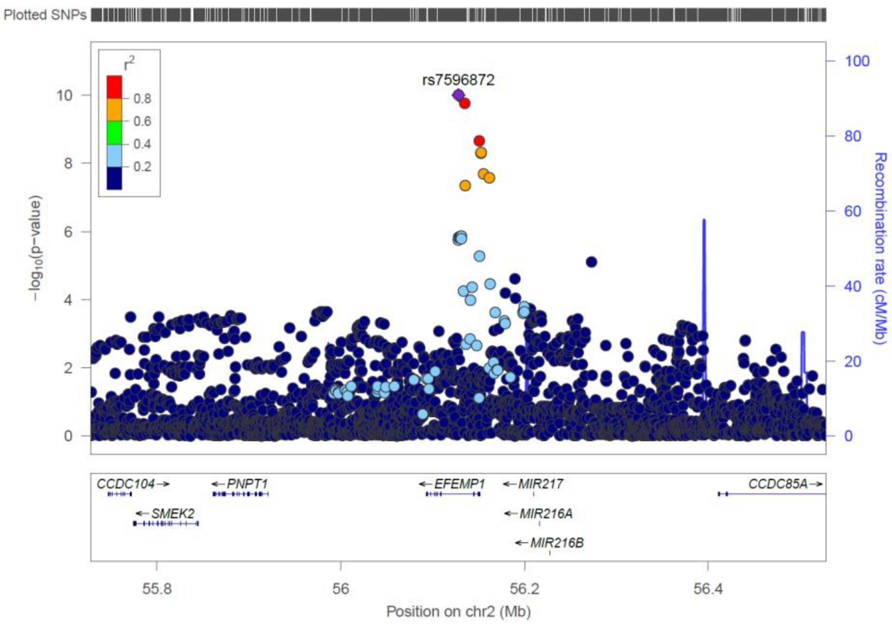

Chr 17q21.31

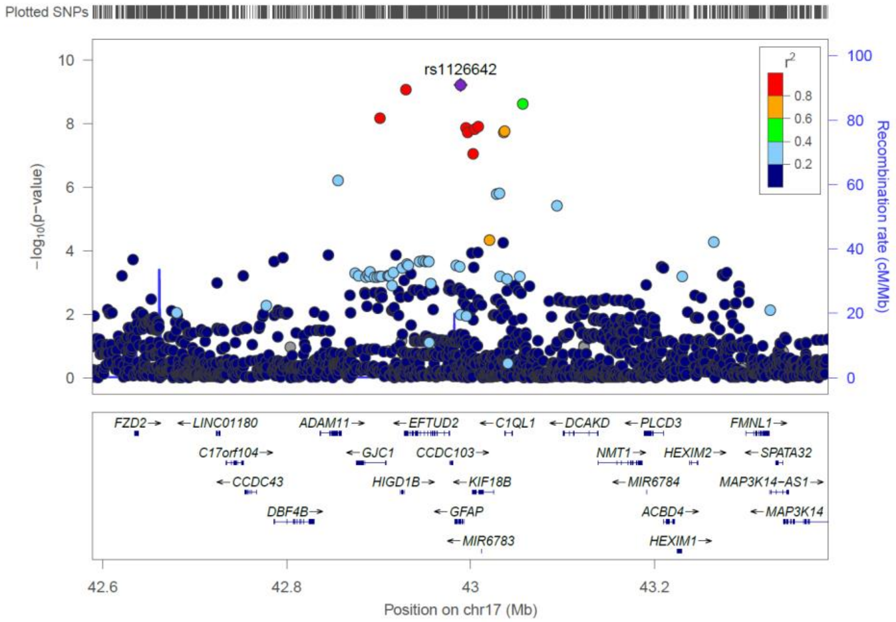

Chr 3q29

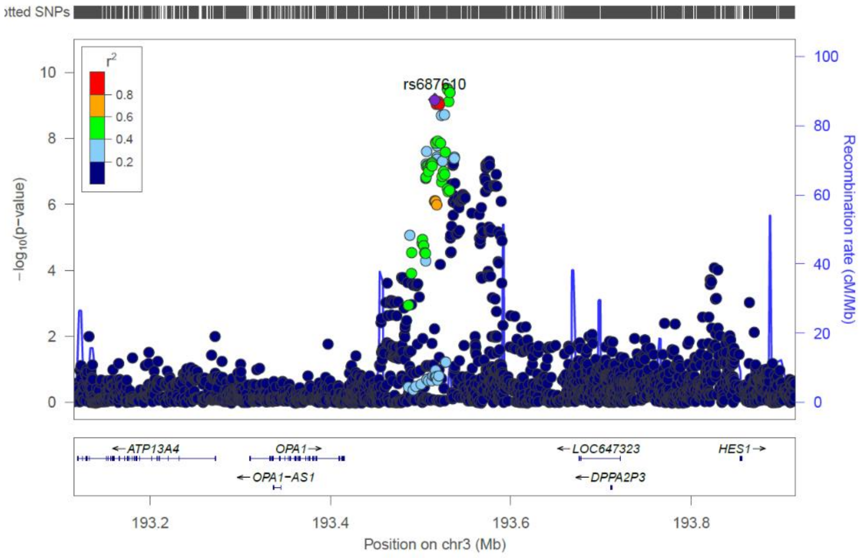

Chr 6p25.2

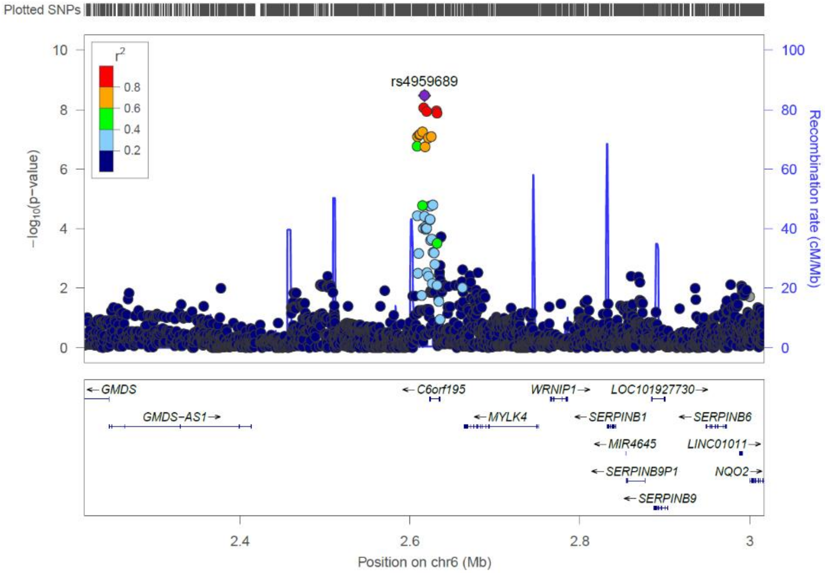

Chr 11q13.3

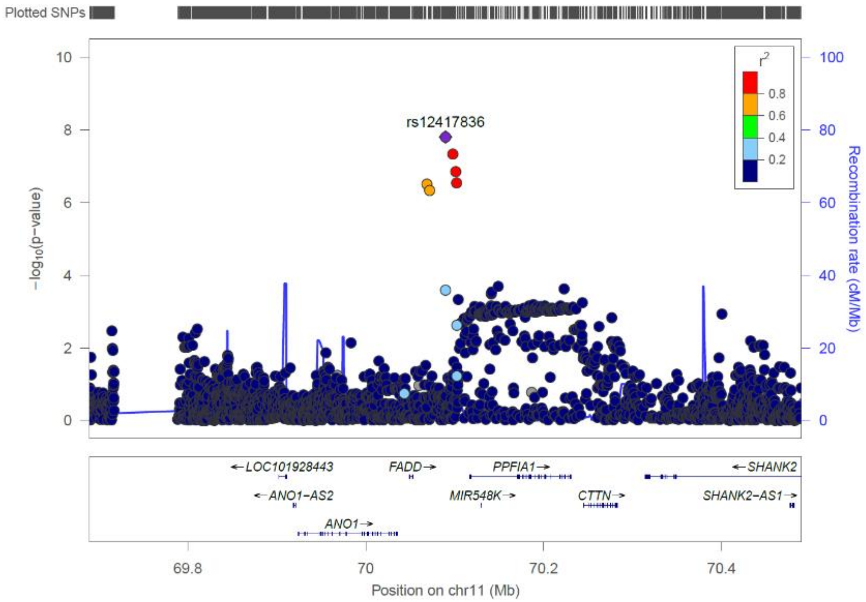

Chr 8p11.21

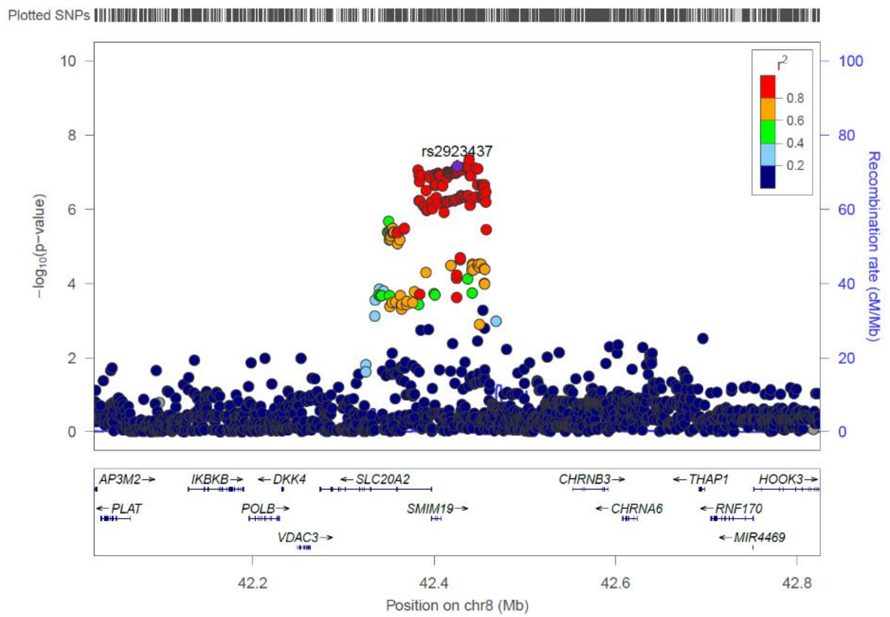

Chr 6p25.3

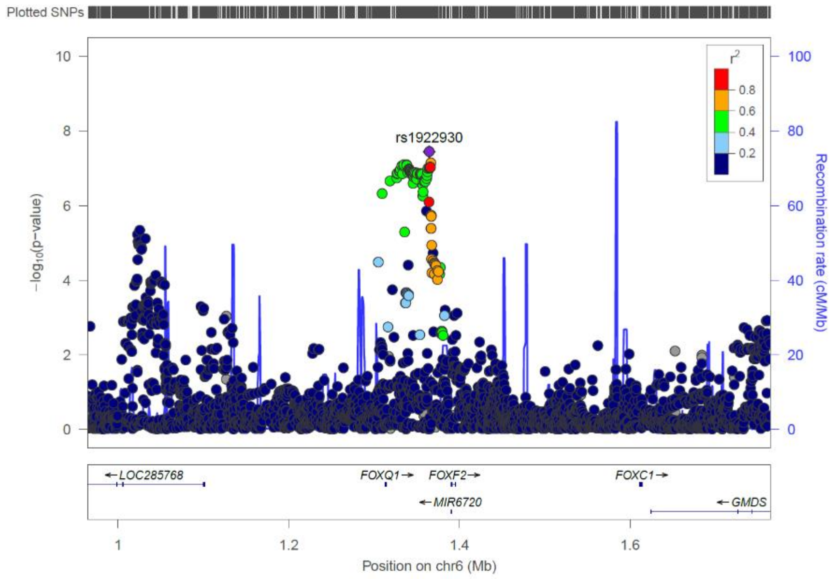

Chr 19p13.11

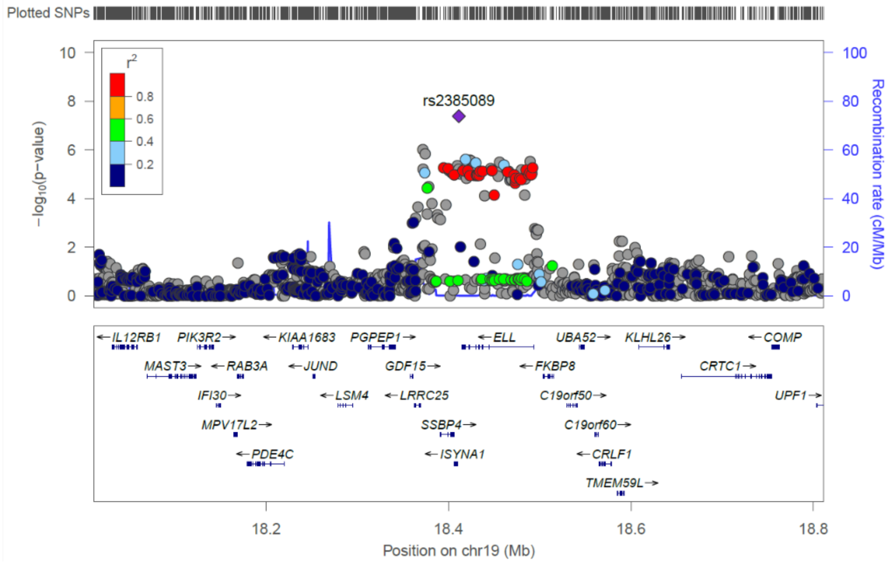

Chr 7q33 (European locus)

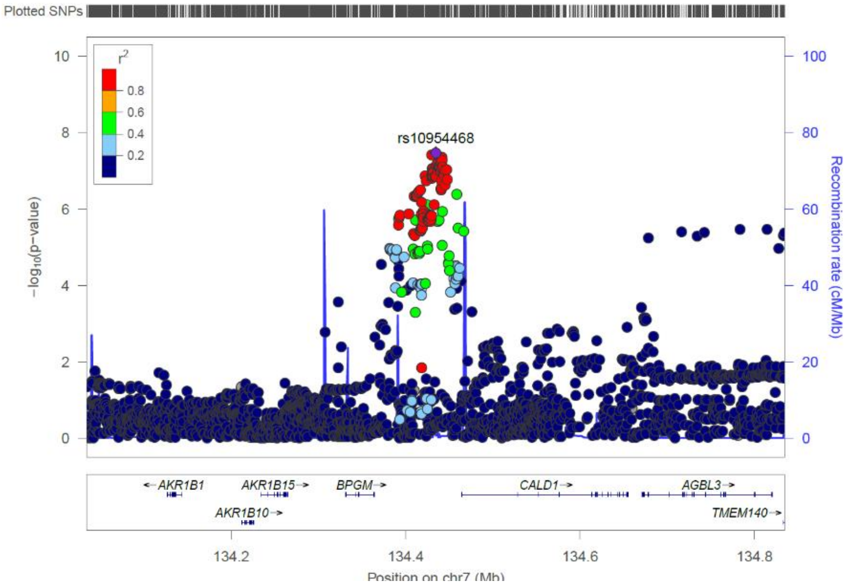

1.2. Perivascular spaces in basal ganglia

Chr2q33.2

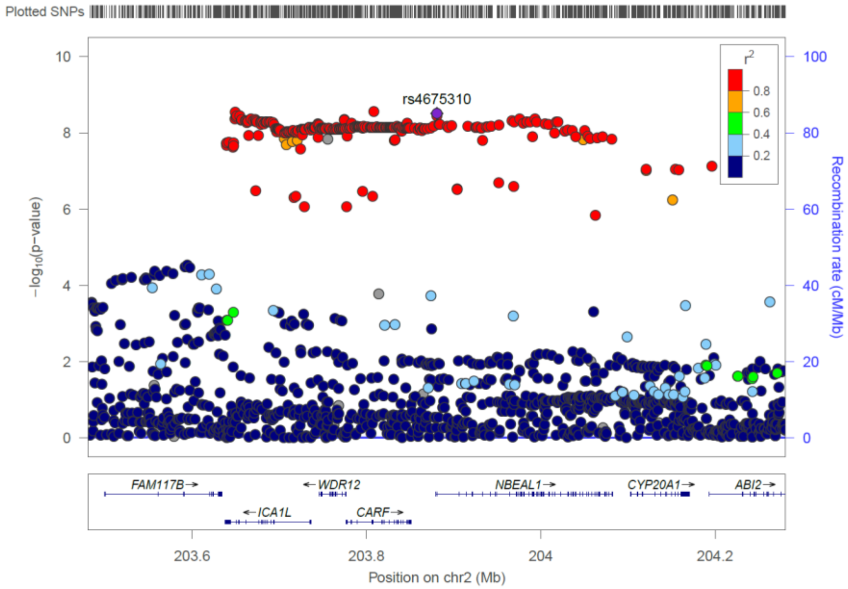

Chr3q26.31

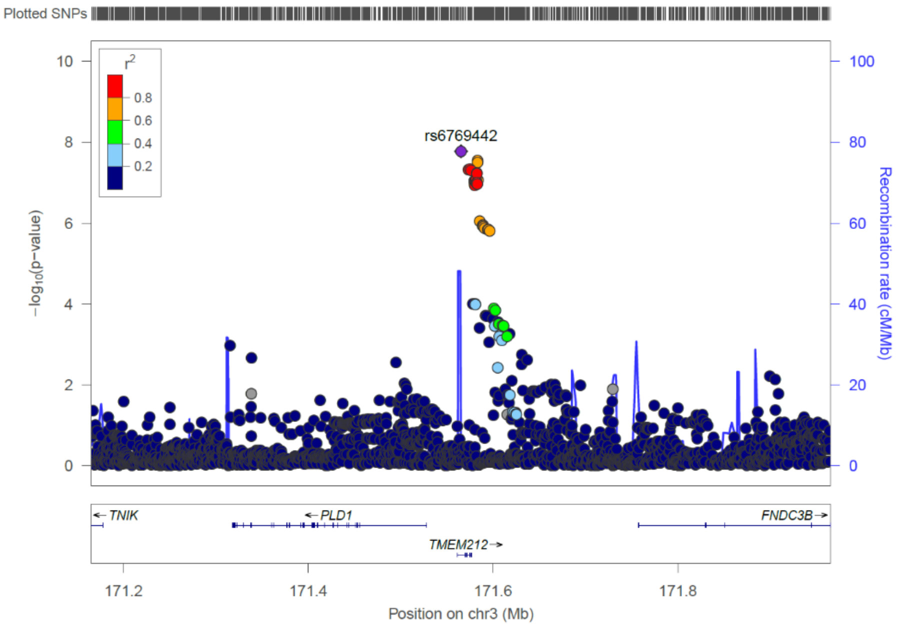

1.3. Perivascular spaces in hippocampus

Chr1q25.3

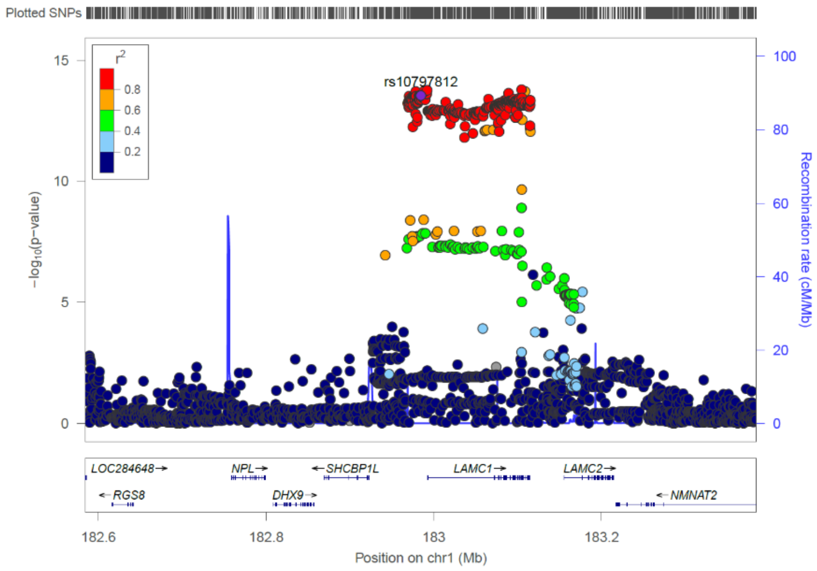

Chr2p16.1

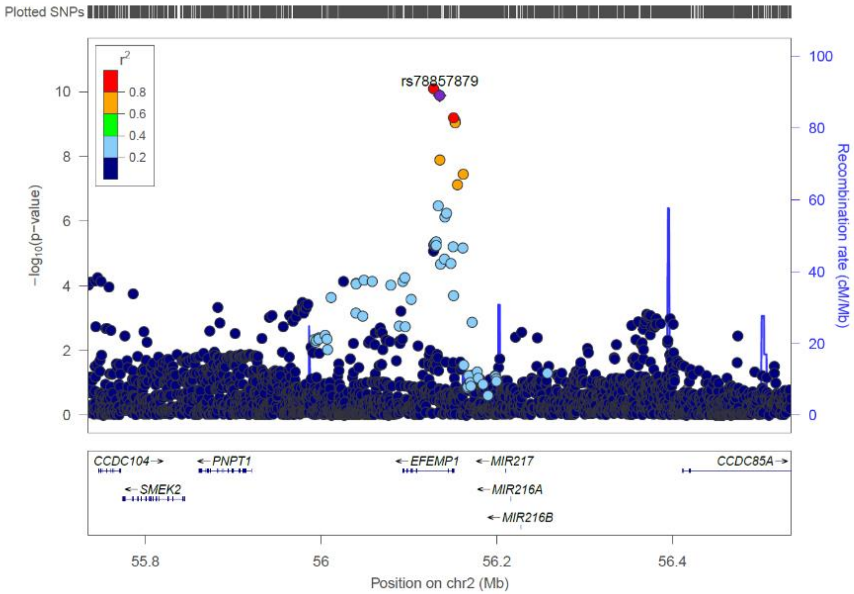

Chr1q41

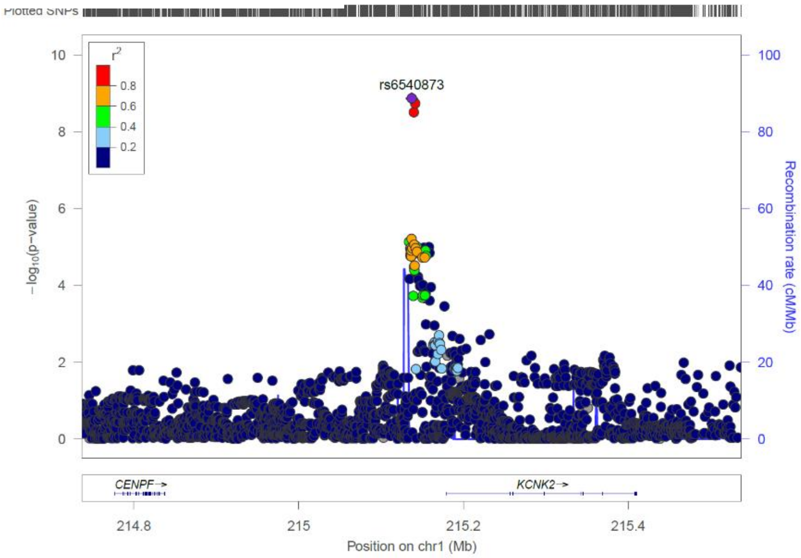

2) **Supplementary Fig. 2 : Association of WM-PVS SNPs with WM-PVS burden in the i-Share cohort (N=1,748)**

A. Continuous PVS measures in i-Share

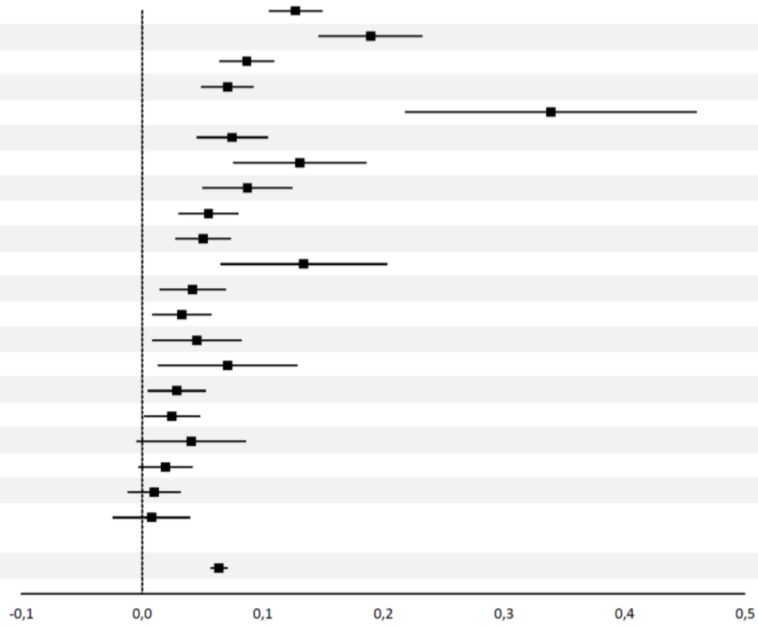

SNPs	β ± SE	p-value
rs687610	0,13 ± 0,022	1,23E-08
rs2425881	0,19 ± 0,043	1,06E-05
rs7596872	0,09 ± 0,022	1,16E-04
rs10494988	0,07 ± 0,022	1,12E-03
rs2425884	0,34 ± 0,121	5,10E-03
rs2385089	0,07 ± 0,029	1,13E-02
rs4685022	0,13 ± 0,055	1,81E-02
rs112407396	0,09 ± 0,037	1,97E-02
rs3772833	0,06 ± 0,025	2,56E-02
rs4959689	0,05 ± 0,023	2,67E-02
rs13079464	0,13 ± 0,069	5,22E-02
rs6011998	0,04 ± 0,027	1,26E-01
rs8041189	0,03 ± 0,024	1,75E-01
rs1126642	0,05 ± 0,037	2,19E-01
rs10817108	0,07 ± 0,058	2,21E-01
rs1922930	0,03 ± 0,024	2,28E-01
rs56104388	0,02 ± 0,023	2,85E-01
rs10954468	0,04 ± 0,045	3,68E-01
rs72485816	0,02 ± 0,023	3,90E-01
rs2923437	0,01 ± 0,022	6,48E-01
rs12417836	0,01 ± 0,032	8,07E-01
rwGRS	0,06 ± 0,007	2,06E-19

B. Dichotomous PVS measures in i-Share

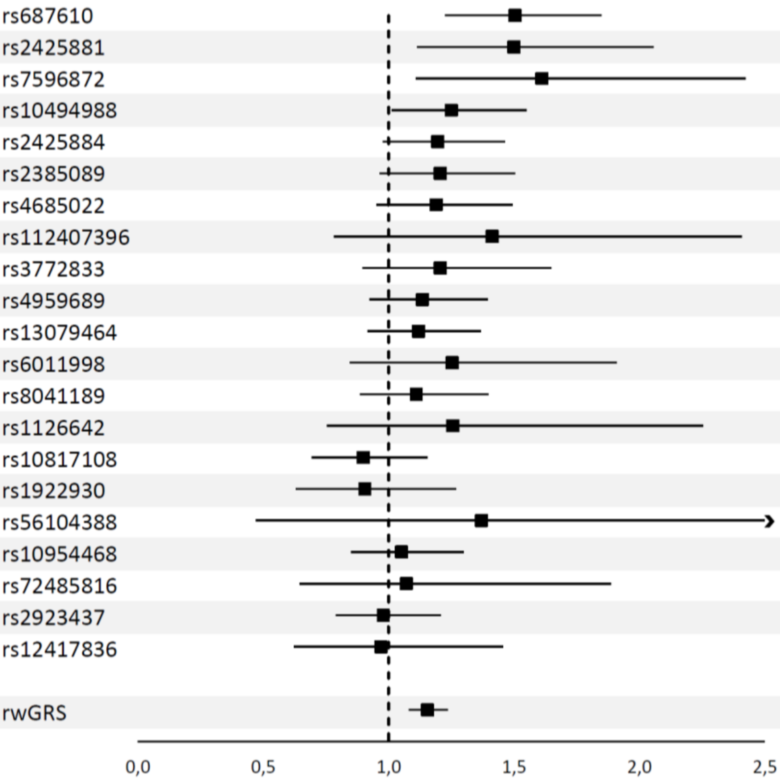

SNP	OR	IC 95%	
rs687610	1,50	1,23	1,85
rs2425881	1,50	1,11	2,06
rs7596872	1,61	1,11	2,42
rs10494988	1,25	1,01	1,55
rs2425884	1,20	0,98	1,46
rs2385089	1,21	0,96	1,50
rs4685022	1,19	0,95	1,49
rs112407396	1,41	0,78	2,41
rs3772833	1,21	0,90	1,65
rs4959689	1,13	0,92	1,40
rs13079464	1,12	0,92	1,37
rs6011998	1,25	0,85	1,91
rs8041189	1,11	0,89	1,40
rs1126642	1,26	0,75	2,25
rs10817108	0,90	0,69	1,16
rs1922930	0,91	0,63	1,27
rs56104388	1,37	0,47	5,82
rs10954468	1,05	0,85	1,30
rs72485816	1,07	0,65	1,89
rs2923437	0,98	0,79	1,21
rs12417836	0,97	0,62	1,46
rwGRS	1,16	1,08	1,24

rwGRS indicates rescaled weighted genetic risk score. Data are presented as effect estimates (beta) +/- SE (A) and as odds Ratio and 95% confidence interval (B). The model is a logistic regression adjusted for age, sex, intracranial volume and the first four principal components of population stratification.

3) Supplementary Fig. 3 : Meta-regression with age of the three genome-wide significant WM-PVS loci that also showed significant associations with WM-PVS in young adults

A. Meta-regression including all the cohorts

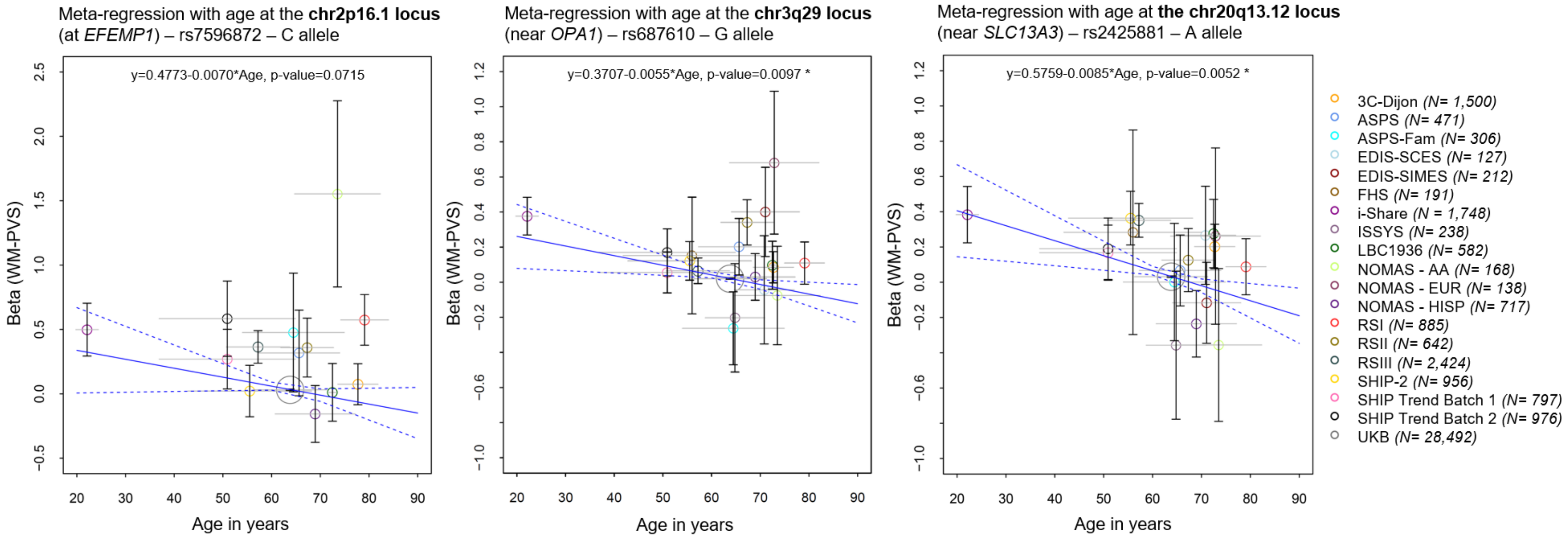

B. Meta-regression without the i-Share Study

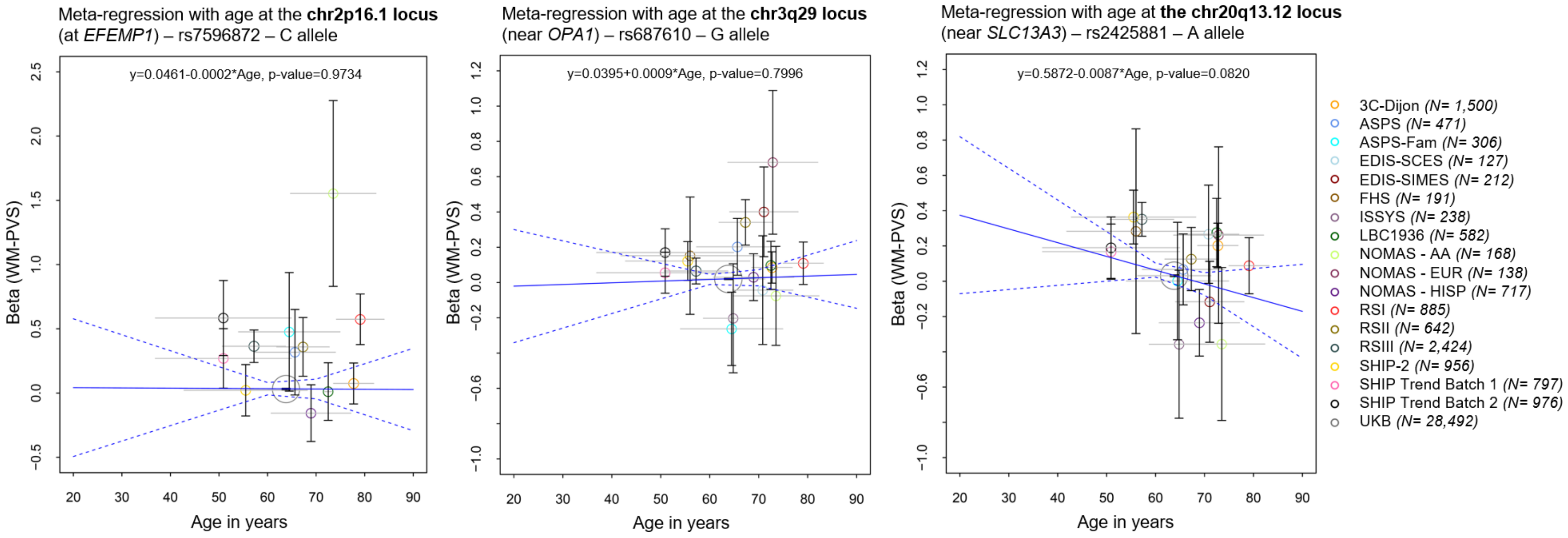

We tested for a significant modifying effect of age on associations with WM-PVS for the three genome-wide significant WM-PVS loci that also showed significant associations with WM-PVS in young adults (Methods). Data are presented as effect estimates (beta) +/- SE for the lead SNPs at these three loci in each individual cohort: A: all cohorts included in the GWAS meta-analysis and the follow-up cohort in young adults (i-Share); B: all cohorts included in the GWAS meta-analysis. *Significant associations (p<0.05/3=0.017, two-sided, correcting for multiple testing).

4) Supplementary Figure 4. Brain expression pattern across the lifespan of genes near genome-wide significant PVS loci that are also identified in the TWAS or that are the nearest genes of variants showing an association with PVS already in young adults

4.1. Genes in WM-PVS loci

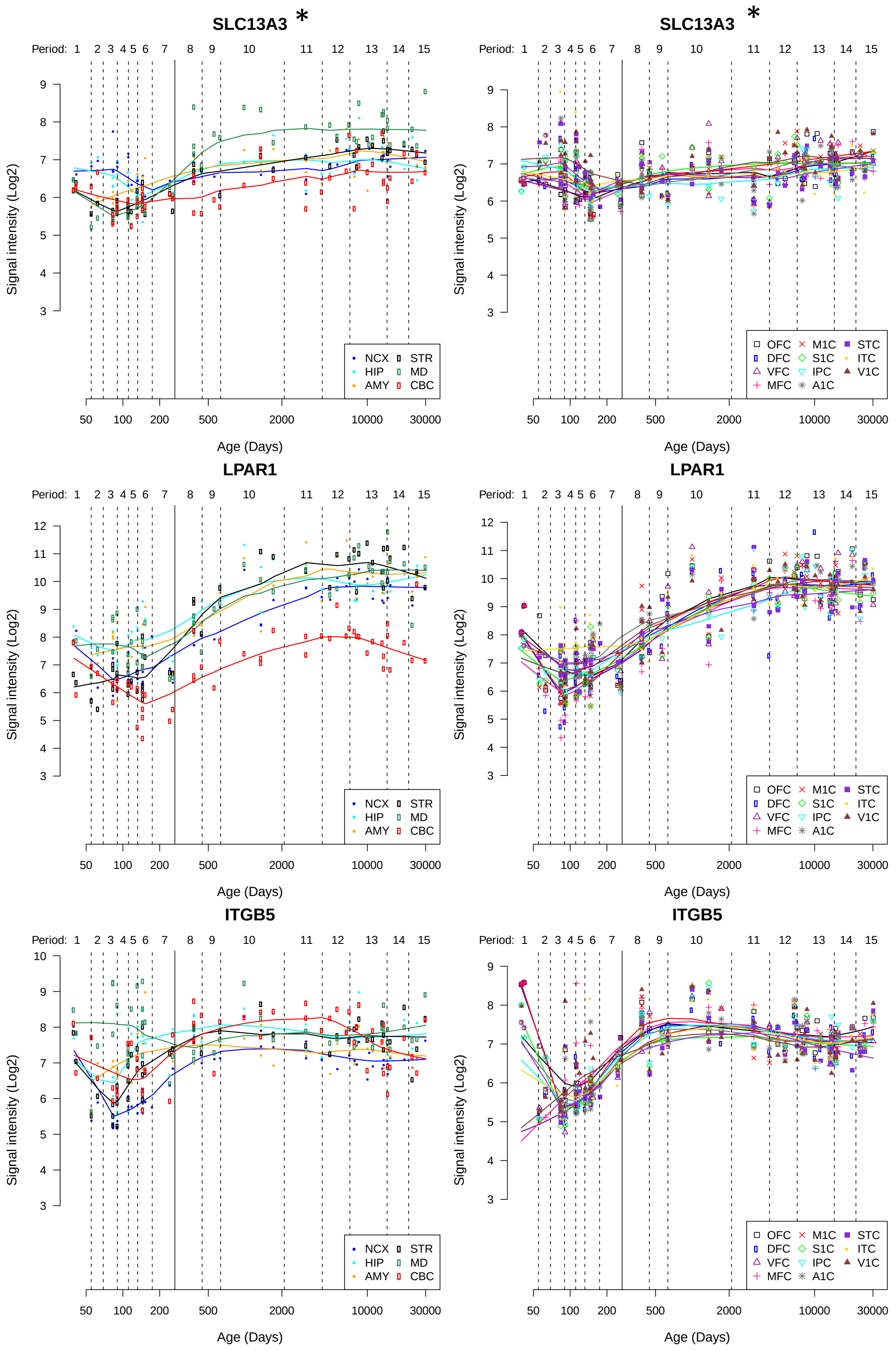

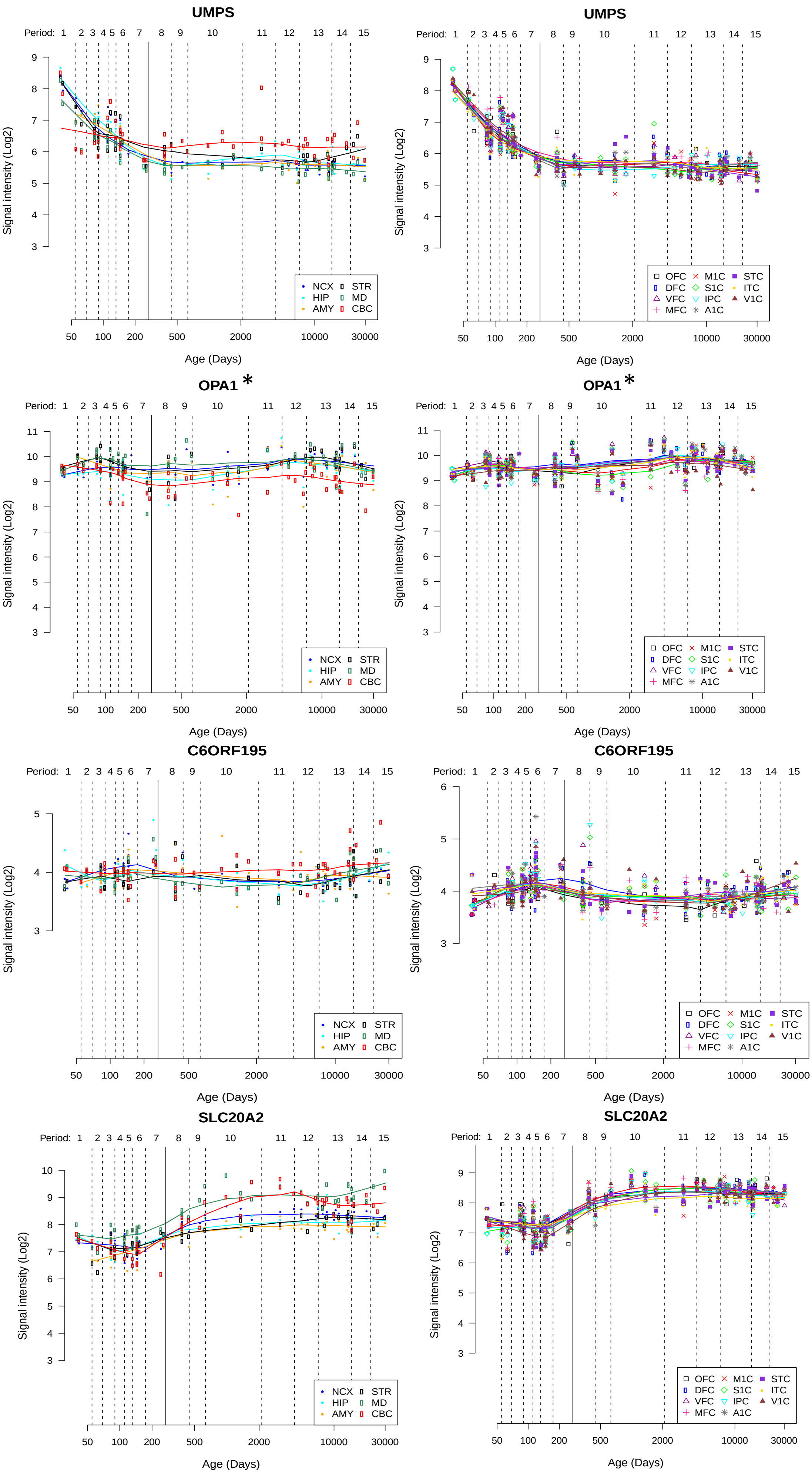
UMPS
Period: 1 2 3 4 5 6 7 8 9 10 11 12 13 14 15
Signal intensity (Log2)
Age (Days)
NCX HIP AMY STR MD CBC
UMPS
OFC DFC VFC MFC M1C S1C IPC A1C STC ITC V1C
OPA1 *
OPA1 *
C6ORF195
C6ORF195
SLC20A2
SLC20A2

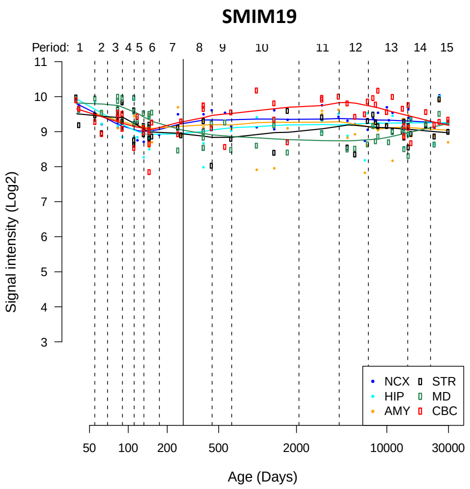

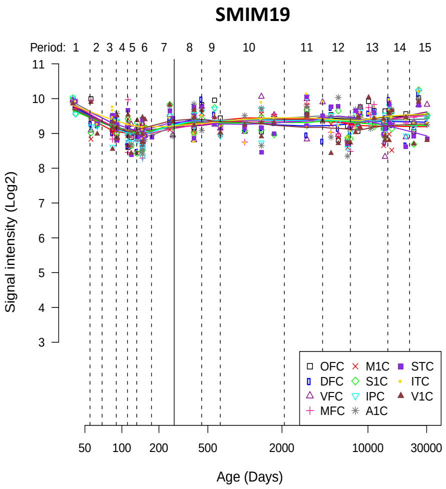

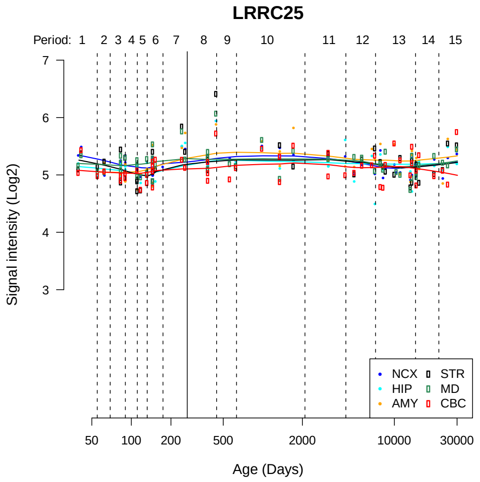

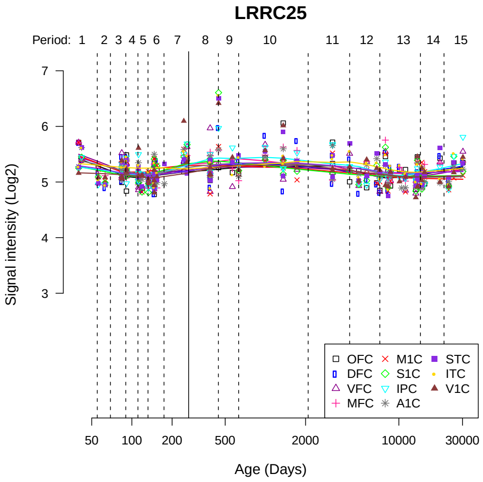

4.2. Genes in BG-PVS loci

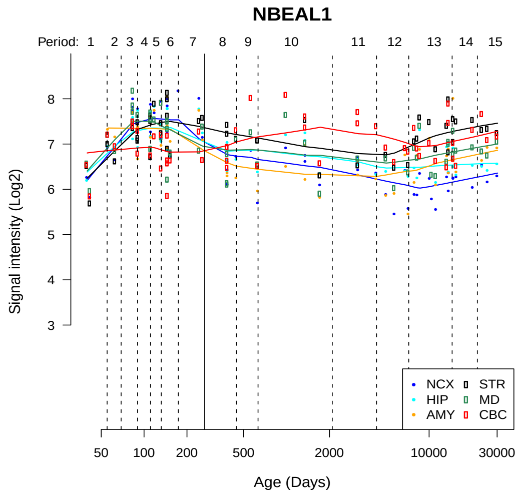

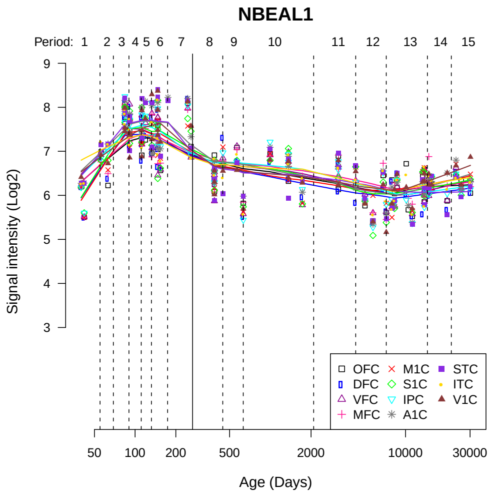

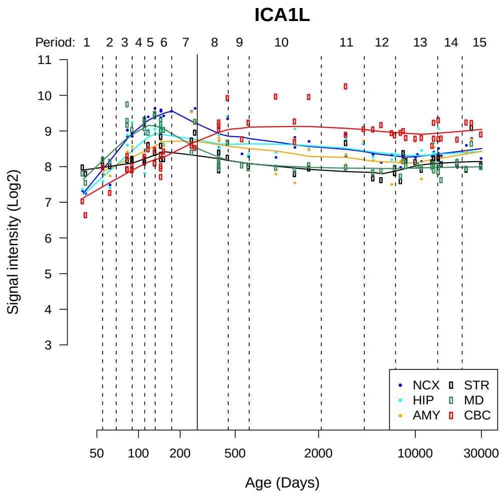

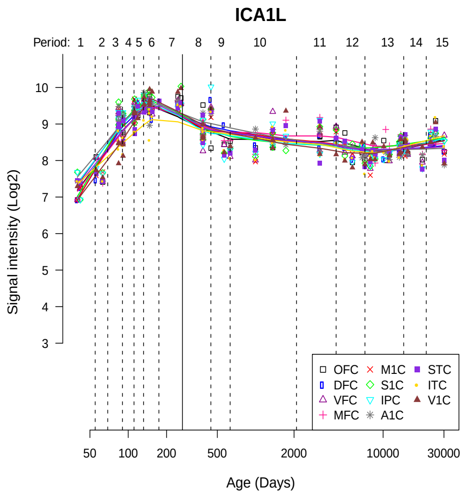

4.3. Genes in HIP-PVS loci

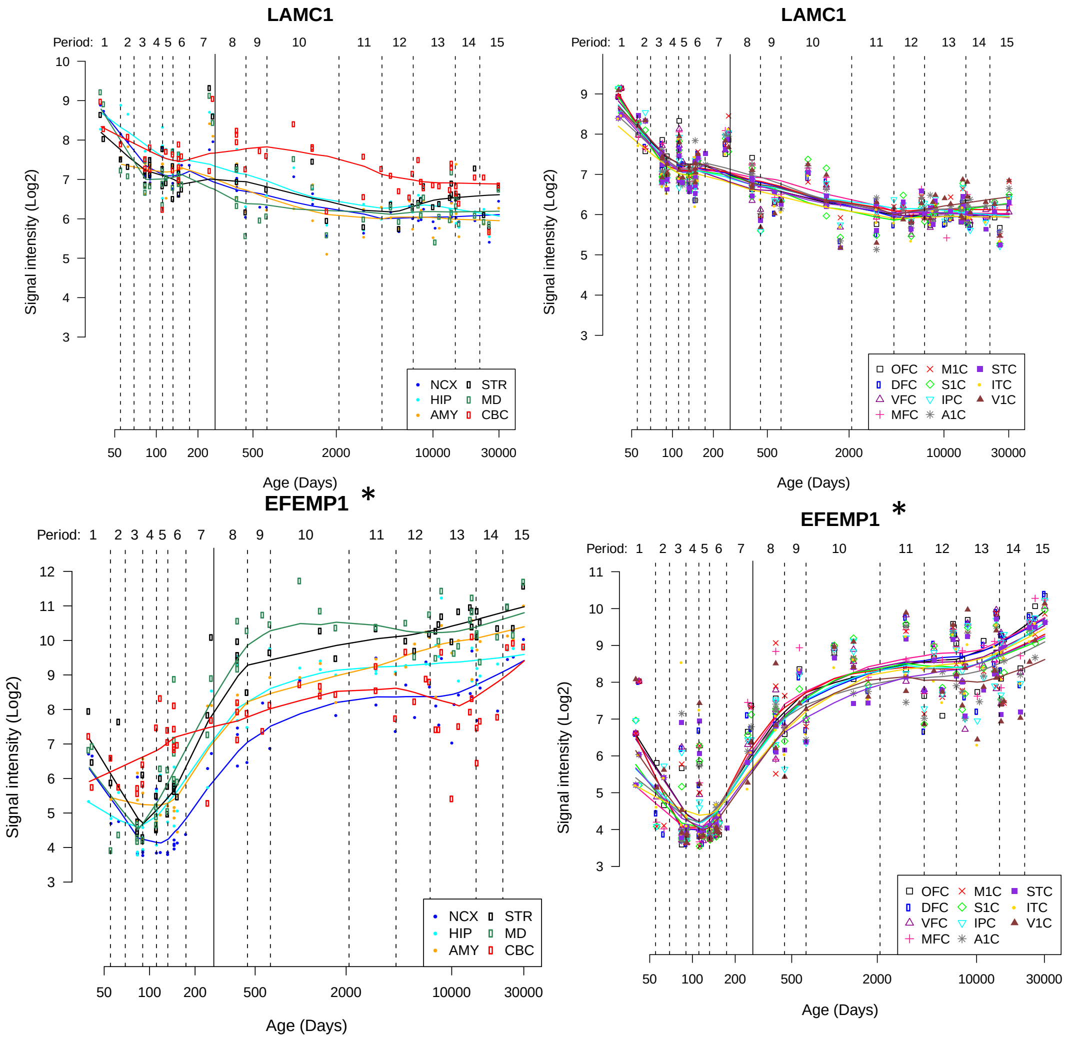

The figure displays brain expression pattern across the lifespan of the 11 significant PVS TWAS-COLOC genes from genome-wide significant PVS GWAS loci and the genes closest to the 3 loci associated with PVS burden across the adult lifespan. The spatio-temporal gene expression level is plotted as log2-transformed exon array signal intensity (y-axis) against the post conception days (x-axis) as provided by the Human Brain Transcriptom project database. Periods of human development and adulthood are indicated by vertical dashed lines: 4-8 post conception weeks [PCW] (period 1), 8-10 PCW (period 2), 10-13 PCW (period 3), 13-16 PCW (period 4), 16-19 PCW (period 5), 19-24 PCW (period 6), 24-38 PCW (period 7), birth- 6 postnatal months (period 8), 6-12 postnatal months (period 9), 1- 6 years (period 10), 6-12 years (period 11), 12-20 years (period 12), 20-40 years (period 13), 40-60 years (period 14), and 60 years+ (period 15). The boundary between pre- and postnatal periods is indicated by the solid vertical line. Each colored point represents the expression level of each gene across 16 anatomical brain regions and ages. Brain structure includes 11 neocortical areas (NCX, blue), and 5 subcortical regions: hippocampus (HIP, cyan), amygdala (AMY, orange), striatum (STR, black), mediodorsal nucleus of thalamus (MD, dark green), and cerebellar cortex (CBC, red).
Neocortical areas include orbital prefrontal cortex (OFC), dorsolateral prefrontal cortex (DFC), ventrolateral prefrontal cortex (VFC), medial prefrontal cortex (MFC), primary motor cortex (M1C), primary somatosensory cortex (S1C), posterior inferior parietal cortex (IPC), primary auditory cortex (A1C), posterior superior temporal cortex (STC), inferior temporal cortex (ITC), and primary visual cortex (V1C). *Genes associated with extensive PVS burden in the GWAS meta-analysis and in the young adult cohort (i-Share study)

5) Supplementary Figure 5. PVS distribution

White matter PVS distribution in the GWAS meta-analysis and the i-Share cohorts

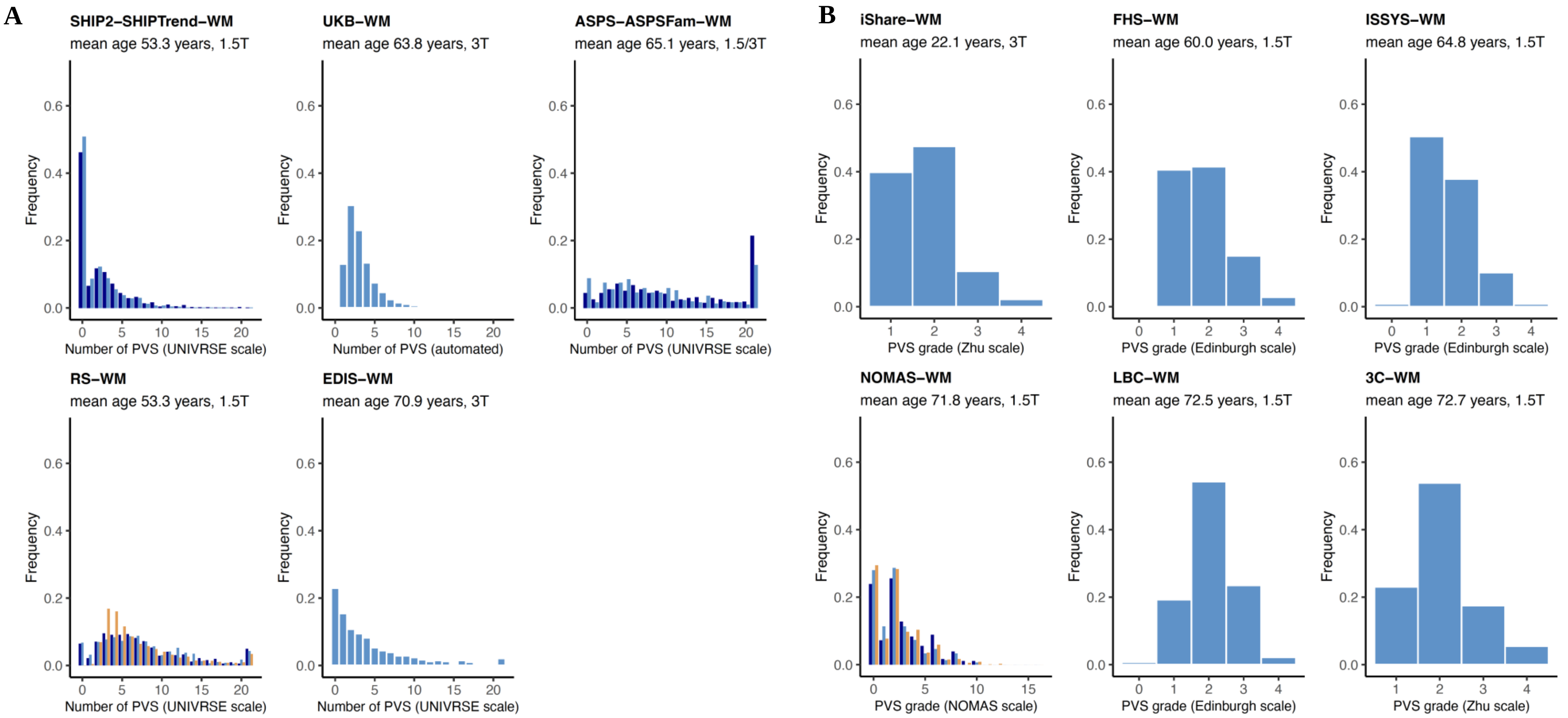

Distribution of PVS burden using the UNIVRSE scale or an automated method (A) and grade-based scales (B). (A) SHIP2 is represented in dark blue, SHIPTrend in light blue; ASPS in dark blue, ASPS-fam in light blue; RSI in dark blue, RS2 in light blue, RS3 in orange. (B) NOMAS-AA (American African ancestry) is represented in dark blue; NOMAS-EUR (European ancestry) in light blue and NOMAS-HISP (Hispanic ancestry) in orange.

Basal ganglia PVS distribution in the GWAS meta-analysis cohorts

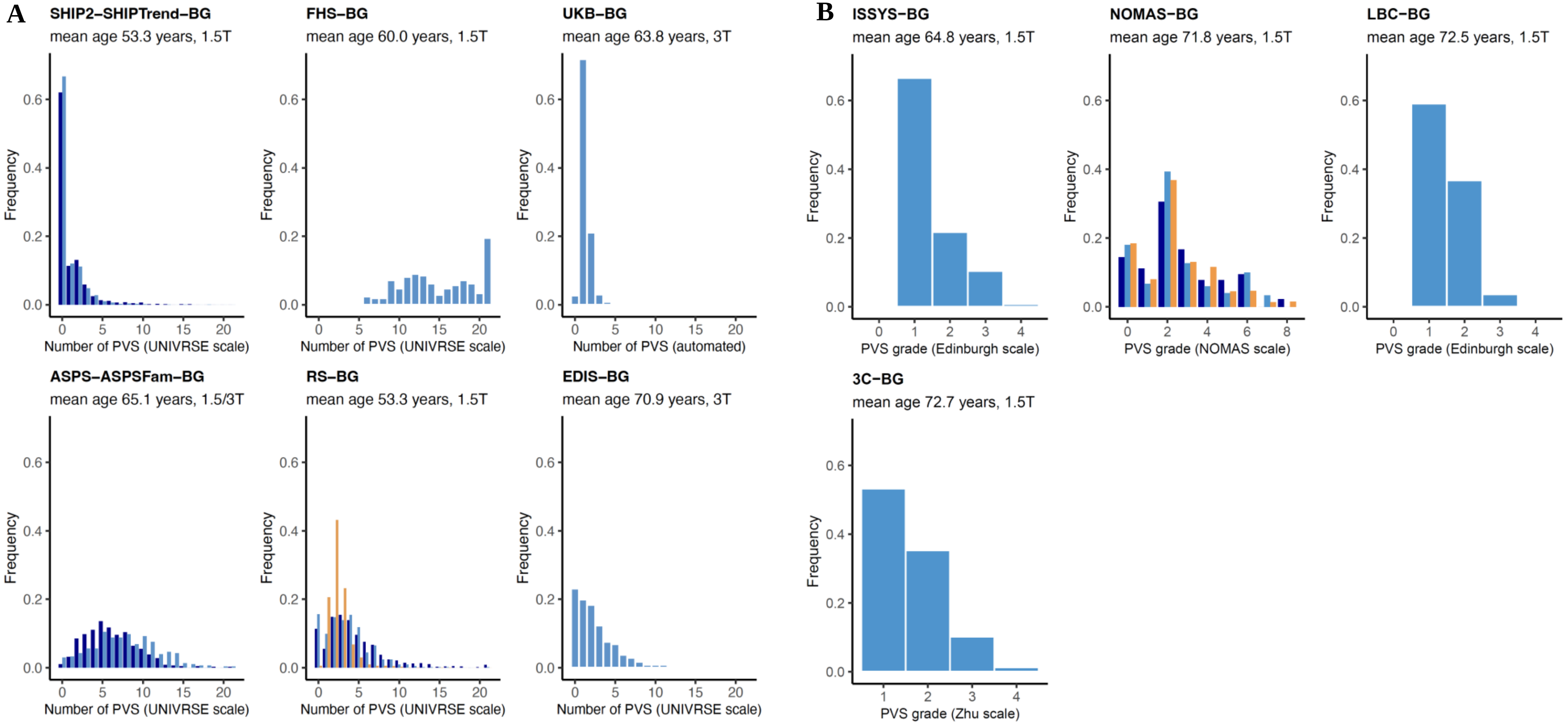

Distribution of PVS burden using the UNIVRSE scale or an automated method (A) and grade-based scales (B). (A) SHIP2 is represented in dark blue, SHIPTrend in light blue; ASPS in dark blue, ASPS-fam in light blue; RSI in dark blue, RS2 in light blue, RS3 in orange. (B) NOMAS-AA (American African ancestry) is represented in dark blue; NOMAS-EUR (European ancestry) in light blue and NOMAS-HISP (Hispanic ancestry) in orange.

Hippocampus PVS distribution in the GWAS meta-analysis cohorts

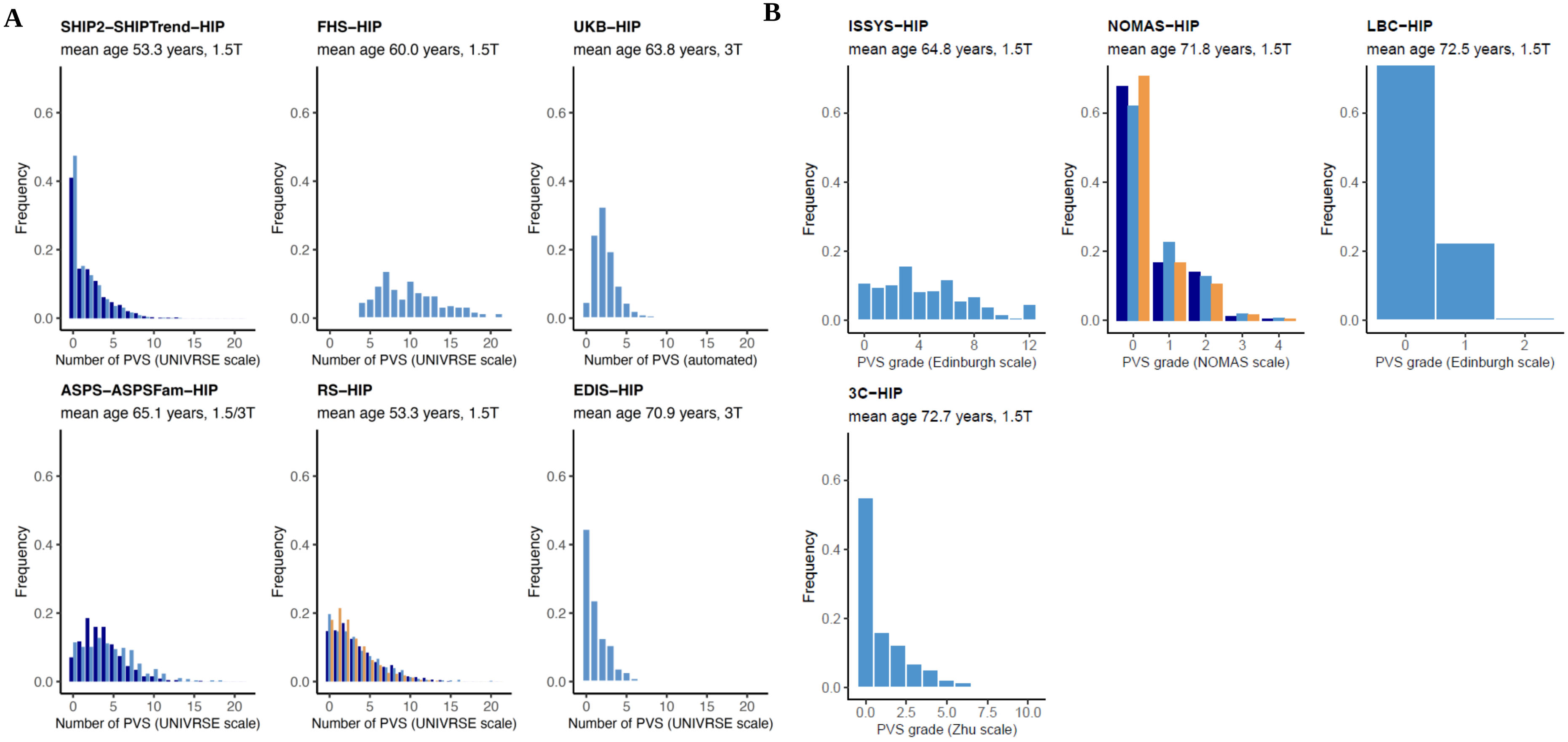

Distribution of PVS burden using the UNIVRSE scale or an automated method (A) and grade-based scales (B). (A) SHIP2 is represented in dark blue, SHIPTrend in light blue; ASPS in dark blue, ASPS-fam in light blue; RSI in dark blue, RS2 in light blue, RS3 in orange. (B) NOMAS-AA (American African ancestry) is represented in dark blue; NOMAS-EUR (European ancestry) in light blue and NOMAS-HISP (Hispanic ancestry) in orange.
