# Supplementary material for: Genomics of perivascular space burden unravels early mechanisms of cerebral small vessel disease
Source: Nat Med. 2023 Apr 17;29(4):950–62. doi: 10.1038/s41591-023-02268-w (PMC10115645; doi:10.1038/s41591-023-02268-w)
Supplement: Supplementary file 2 — Reporting Summary [file 41591_2023_2268_MOESM2_ESM.pdf]

Corresponding author(s): Hieab Adams, Stéphanie Debette

Last updated by author(s): Feb 7, 2023

Reporting Summary

Nature Portfolio wishes to improve the reproducibility of the work that we publish. This form provides structure for consistency and transparency in reporting. For further information on Nature Portfolio policies, see our Editorial Policies and the Editorial Policy Checklist.

Statistics

For all statistical analyses, confirm that the following items are present in the figure legend, table legend, main text, or Methods section.

n/a	Confirmed	
☐	☒	The exact sample size (*n*) for each experimental group/condition, given as a discrete number and unit of measurement
☐	☒	A statement on whether measurements were taken from distinct samples or whether the same sample was measured repeatedly
☐	☒	The statistical test(s) used AND whether they are one- or two-sided<br>*Only common tests should be described solely by name; describe more complex techniques in the Methods section.*
☐	☒	A description of all covariates tested
☐	☒	A description of any assumptions or corrections, such as tests of normality and adjustment for multiple comparisons
☐	☒	A full description of the statistical parameters including central tendency (e.g. means) or other basic estimates (e.g. regression coefficient) AND variation (e.g. standard deviation) or associated estimates of uncertainty (e.g. confidence intervals)
☐	☒	For null hypothesis testing, the test statistic (e.g. *F*, *t*, *r*) with confidence intervals, effect sizes, degrees of freedom and *P* value noted<br>*Give P values as exact values whenever suitable.*
☒	☐	For Bayesian analysis, information on the choice of priors and Markov chain Monte Carlo settings
☒	☐	For hierarchical and complex designs, identification of the appropriate level for tests and full reporting of outcomes
☐	☒	Estimates of effect sizes (e.g. Cohen's *d*, Pearson's *r*), indicating how they were calculated

Our web collection on statistics for biologists contains articles on many of the points above.

Software and code

Policy information about availability of computer code

Data collection	No software was used for data collection.
Data analysis	EasyQC 9.0, METAL/2011-03-25, GCTA 1.93.0 (GCTA-COJO and GSMR), MR-MEGA vO.1.5, LD Score Regression version1.0.0, MTAG version1.0.8, Metafor package 1.9-9 (R version 3.6.1), R 3.6.1 and R 4.0.2 (Mendelian randomization and TWAS-fusion packages), FUMA v1.3.5e, VEGAS2 version 2.01.17, COSMIC v87, COLOC R package (version5), STEAP pipeline v1, GREP v1.0.0, Trans-Phar v1, REGENIE v2.0.1, RVtest, Plink 1.9

For manuscripts utilizing custom algorithms or software that are central to the research but not yet described in published literature, software must be made available to editors and reviewers. We strongly encourage code deposition in a community repository (e.g. GitHub). See the Nature Portfolio guidelines for submitting code & software for further information.

Data

Policy information about availability of data

All manuscripts must include a data availability statement. This statement should provide the following information, where applicable:

- Accession codes, unique identifiers, or web links for publicly available datasets
- A description of any restrictions on data availability
- For clinical datasets or third party data, please ensure that the statement adheres to our policy

Genome-wide summary statistics for the European and cross-ancestry meta-analysis generated and analyzed during the current study are deposited on the GWAS

Catalog (study code GCST90244151-GCST90244156). As for other meta-analyses of GWAS or sequencing data, individual cohort data are subject to controlled access, for privacy and legal issues (national and European regulations, including GDPR). This applies to all participating cohorts (cohorts included in the meta-analyses and follow-up cohorts). UKB data (GWAS and sequencing) is accessible by submitting an application to the UKB portal (This research has been conducted under Application Number 23509). We used publicly available data from GTEx (https://gtexportal.org/home/), the Gusev laboratory (http://gusevlab.org/projects/fusion/), the CommonMind Consortium (https://www.nimhgenetics.org/resources/commonmind), the Netherlands Twin Registry (https://tweelingenregister.vu.nl/), the Young Finns Study (https://youngfinnsstudy.utu.fi/), OMIM (https://www.omim.org/), OMIM genes description are publicly available: GFAP (https://www.omim.org/entry/137780); SLC13A3 (https://www.omim.org/entry/618384); PNPT1 (https://www.omim.org/entry/610316), COSMIC (https://cancer.sanger.ac.uk), RNA-seq datasets: PsychENCODE DER-22 (www.ncbi.nlm.nih.gov/geo/, accession code GSE97942), GSE67835 (www.ncbi.nlm.nih.gov/geo/, accession code GSE67835), GSE101601 (https://www-ncbi-nlm-nih-gov.proxy.insermbiblio.inist.fr/geo/query/acc.cgi?acc=GSE101601), DroNc_Human Hippocampus (https://www.gtexportal.org/home/datasets), Allen Brain Atlas (http://portal.brain-map.org/), Descartes_Human (https://descartes.brotmanbaty.org/), Mousebrain (http://mousebrain.org/), Tabula Muris (https://tabula-muris.ds.czbiohub.org/). All other data supporting the findings of this study are available either within the article, the supplementary information and supplementary data files.

Human research participants

Policy information about studies involving human research participants and Sex and Gender in Research.

Reporting on sex and gender	Sex was genetically determined. While we had limited power to run sex-specific analyses with the dichotomized PVS variables, with increasing development of AI-based computational methods for PVS quantification, future genomic studies will have greater power for such important explorations.
Population characteristics	Up to 40,095 participants (38,871 Europeans, 717 Hispanics, 339 East-Asians and 168 African-Americans) were included in the GWAS meta-analysis (66.3±8.6 years, 51.7% female, 66.7% with hypertension). Ancestry-specific logistic regression analyses with an additive genetic model were performed, adjusting for age, sex (genetically determined), and intracranial volume (or brain parenchymal fraction for ASPS), relevant principal components of population stratification, and study site. Ancestry was genetically inferred using principal component analyses. Detailed population characteristics by study and phenotype definitions can be found in the Supplementary Methods and in Supplementary Table 1. Detailed population characteristics for the replication and whole genome / whole exome sequencing analyses can be found in Supplementary Table 1.
Recruitment	All participating studies had population-based recruitment strategies. Details can be found in the Supplementary Methods.
Ethics oversight	Study protocols were approved for all studies by the appropriate boards at their respective institutions. Details can be found in the Supplementary Methods and in the Supplementary Table 1.

Note that full information on the approval of the study protocol must also be provided in the manuscript.

Field-specific reporting

Please select the one below that is the best fit for your research. If you are not sure, read the appropriate sections before making your selection.

☒ Life sciences ☐ Behavioural & social sciences ☐ Ecological, evolutionary & environmental sciences

For a reference copy of the document with all sections, see nature.com/documents/nr-reporting-summary-flat.pdf

Life sciences study design

All studies must disclose on these points even when the disclosure is negative.

Sample size	We conducted a cross-ancestry genome-wide association (GWAS) of perivascular space burden (PVS) in up to 40,095 participants. This sample size corresponds to the largest available sample of population-based study participants with PVS measurements and genome-wide genotype data that we were able to gather (this is the same order of magnitude of other recent published GWAS on MRI-markers of brain aging; Methods - page 1, Supplementary Table 1 and Supplementary Methods - pages 4-8 for more information about the samples). With increasing development of AI-based computational methods for PVS quantification, future genomic studies will likely have even greater power to detect genetic associations, to enable studying the genomics of total PVS volume, accounting for differences in individual PVS volume, width, length, shape,60 density, location, anatomical predominance and to run sex-specific analyses.
Data exclusions	Participants were excluded from the analysis if they had co-morbidities modifying the measurement of the phenotype (stroke at time of MRI and brain tumor or other condition that may bias PVS measurement), missing covariates (Supplementary Table 1). Genotyped or imputed variants were excluded if they failed quality control filters described in the Methods (pages 3-4).
Replication	We conducted a GWAS meta-analysis in 18 population-based cohorts. We replicated the genome-wide significant associations in the independant Nagahama sample (N=2,862) and i-Share (N=1,748).
Randomization	We did not allocate participants into experimental groups. However we did conduct Mendelian randomization analyses based on genetic associations in the study population (described in the Methods).
Blinding	Not relevant for the present study design (strictly speaking PVS burden was measured blinded to the genotypes and vice versa). The consortium meta-analyzed summary statistics from case/control studies, not individual level data.

Reporting for specific materials, systems and methods

We require information from authors about some types of materials, experimental systems and methods used in many studies. Here, indicate whether each material, system or method listed is relevant to your study. If you are not sure if a list item applies to your research, read the appropriate section before selecting a response.

Materials & experimental systems

n/a	Involved in the study
☒	☐ Antibodies
☒	☐ Eukaryotic cell lines
☒	☐ Palaeontology and archaeology
☒	☐ Animals and other organisms
☒	☐ Clinical data
☒	☐ Dual use research of concern

Methods

n/a	Involved in the study
☒	☐ ChIP-seq
☒	☐ Flow cytometry
☐	☒ MRI-based neuroimaging

Magnetic resonance imaging

Experimental design

Design type: Population-based brain imaging study.

Design specifications: The recruitment strategy for each cohort studies is described in the Supplementary Methods (section 1).

Behavioral performance measures: There are no behavioral performance measures in this study.

Acquisition

Imaging type(s): Brain MRI with T1-, T2-weighted, FLAIR and proton density sequences images. Study-specific information is described in Supplementary Methods (section 1-3).

Field strength: 1.5 and 3.0 Tesla.

Sequence & imaging parameters: Study-specific sequence and imaging parameters information is described in the Supplementary Methods (Section 3).

Area of acquisition: Brain.

Diffusion MRI ☐ Used ☒ Not used

Preprocessing

Preprocessing software: Study-specific information is described in the Supplementary Methods (Section 1 and 3).

Normalization: Study-specific information is described in the Supplementary Methods (Section 1 and 3).

Normalization template: Study-specific information is described in the Supplementary Methods (Section 1 and 3).

Noise and artifact removal: Study-specific information is described in the Supplementary Methods (Section 1 and 3).

Volume censoring: Study-specific information is described in the Supplementary Methods (Section 1 and 3).

Statistical modeling & inference

Model type and settings: Detailed information on the models tested are provided in the Methods (page 1-14).

Effect(s) tested: Association of genetic variants with perivascular space burden in white matter, basal ganglia and hippoampus.

Specify type of analysis: ☒ Whole brain ☐ ROI-based ☐ Both

Statistic type for inference (See Eklund et al. 2016): Non relevant for this study.

Correction: Bonferroni

Models & analysis

n/a	Involved in the study
☒	☐ Functional and/or effective connectivity
☒	☐ Graph analysis
☒	☐ Multivariate modeling or predictive analysis
